# Novel dual gland GAN architecture improves human protein localization classification using salivary and pituitary gland inspired loss functions

**DOI:** 10.1038/s41598-025-11254-w

**Published:** 2025-08-01

**Authors:** Hanaa Salem Marie, Moatasem M. Draz, Waleed Abd Elkhalik, Mostafa Elbaz

**Affiliations:** 1 Faculty of Artificial Intelligence, Delta University for Science and Technology, Gamasa, 35712 Egypt; 2 Information Systems Department, Faculty of Computers and Information, Kafrelsheikh University, Kafrelsheikh, Egypt; 3 Lecturer at Faculty of Computers and Informatics, Tanta University, Tanta, Egypt; 4 Department of Computer Science, Faculty of Computers and Informatics, Kafrelsheikh University, Kafrelsheikh, Egypt

**Keywords:** Cellular classification, Human protein atlas, Bioimage informatics, Protein localization, Cell phenotyping, Cell delivery, Stem-cell biotechnology

## Abstract

Cellular classification is essential for understanding biological processes and disease mechanisms. This paper introduces a novel approach that employs two complementary loss functions within a Generative Adversarial Network (GAN) framework for processing images from the Human Protein Atlas dataset. Our method introduces the “Salivary Gland” loss function (SG-Loss), which addresses missing pixel imputation through a unique computational mechanism that models the graded secretion patterns of acinar cells, incorporating multi-scale contextual information to reconstruct incomplete cellular features. This is paired with our innovative “Pituitary Gland” loss function (PG-Loss), which preserves structural integrity through a novel homeostatic regularization approach that adaptively weights pixel relationships based on subcellular compartment boundaries, unlike conventional smoothing techniques. The SG-Loss specifically targets discontinuities in protein expression patterns, while PG-Loss maintains biological plausibility by enforcing organelle-specific constraints learned from annotated training data. Our proposed Dual-Gland GAN demonstrates superior performance with an Inception Score of 9.83 (± 0.31) and MS-SSIM Diversity of 0.187 (± 0.021). The model achieves impressive precision and recall metrics (0.872 and 0.835, respectively), resulting in an F1-score of 0.853. Training stability is reflected in minimal generator and discriminator loss variance (0.028 and 0.032) with convergence achieved in 78 epochs. Comprehensive evaluation shows high quality and diversity scores (0.912 and 0.894), yielding a combined score of 0.903, demonstrating the effectiveness of our biologically inspired approach for cellular image generation and classification. The results also prove the efficiency of the architecture in enhancing the classification results.

## Introduction

The Human Protein Atlas (HPA) is a pioneering resource that provides a comprehensive map of protein expression and localization across various human tissues and cell types^1–3^. Established to facilitate a deeper understanding of human biology, the HPA integrates extensive data derived from multiple experimental techniques, including immunohistochemistry, transcriptomics, and mass spectrometry. This atlas offers a wealth of information, detailing the spatial distribution of proteins within different cellular contexts, and serves as a crucial reference for researchers investigating the roles of proteins in both health and disease. By characterizing protein expression patterns, the HPA enhances our understanding of the molecular underpinnings of various physiological processes and pathological conditions^4,5^. HPA fosters collaborative efforts among researchers worldwide, promoting innovation in fields such as cancer research, neurobiology, and regenerative medicine. The ability to visualize protein expression in situ not only aids in the identification of potential biomarkers for disease but also supports the development of targeted therapies that can be tailored to individual patients based on their unique protein profiles^6^.

Cell-wise classification within the HPA is essential for accurately identifying and characterizing different cell types based on their specific protein expression profiles. This classification is crucial for understanding cellular heterogeneity, as it allows researchers to discern how distinct cell types contribute to overall tissue function and disease processes. Accurate cell classification enhances our ability to investigate cellular interactions, signaling pathways, and the functional roles of proteins, ultimately informing the development of novel therapeutic strategies and personalized medicine approaches^7,8^.

Recent advancements in deep learning methods have revolutionized the landscape of cell-wise classification, enabling more sophisticated analyses of complex biological datasets^9^. Techniques such as convolutional neural networks (CNNs) and transformer architecture have been successfully applied to extract meaningful features from high-dimensional data, resulting in significant improvements in classification accuracy and robustness^10^. These methodologies facilitate the extraction of intricate patterns in protein expression and localization, thereby enhancing our understanding of cellular dynamics and their implications in health and disease.

Despite the extensive amount of information provided by the Human Protein Atlas (HPA), several specific limitations exist within the available datasets that severely impact machine learning model performance. First, the extreme class imbalance problem where nucleoplasm represents 27.4% of samples while rare localizations like mitotic spindle constitute only 1.3%, creating a 21:1 imbalance ratio that leads to biased model predictions favoring dominant classes. Second, the insufficient representation of morphological diversity within each protein localization class, where subtle but biologically significant variations in cellular phenotypes are underrepresented, limiting model generalization to novel cellular contexts. Third, the missing pixel artifacts that frequently occur during high-resolution fluorescence microscopy imaging, particularly at organellar boundaries and in regions with weak protein expression, which create incomplete training examples that degrade classification performance. Fourth, the poor connectivity preservation between neighboring pixels in cellular structures, where traditional augmentation techniques fail to maintain the biological plausibility of organellar networks and protein distribution patterns essential for accurate classification^11,12^.

The primary aim of this paper is to systematically address these four critical limitations through a novel dual-generator GAN architecture that specifically tackles: (1) class imbalance through targeted minority class augmentation, (2) morphological diversity enhancement via biologically-constrained synthetic image generation, (3) missing pixel imputation using secretion-pattern modeling, and (4) connectivity preservation through homeostatic regularization mechanisms. While our methodology is primarily developed and validated on HPA data, the underlying biological principles of cellular organization, protein localization patterns, and organellar structure are universal across mammalian cell types, making our approach broadly applicable to other fluorescence microscopy datasets including immunofluorescence imaging, live-cell imaging, and multi-channel cellular phenotyping studies beyond the HPA domain.

Generative Adversarial Networks (GANs) are a powerful class of machine learning models that consist of two neural networks: a generator and a discriminator^13^. The generator creates synthetic images, while the discriminator evaluates their authenticity against real images. This adversarial process continues until the generator produces images that are indistinguishable from real ones. GANs can be particularly useful for data augmentation, as they can generate diverse and high-quality images that enhance existing datasets. In the context of the Human Protein Atlas (HPA), GANs can help address limitations such as insufficient representation of various cell types and conditions. By generating realistic protein expression images, GANs can enrich the dataset, improve the training of machine learning models, and ultimately lead to more accurate classifications and insights into protein localization and function^14,15^.

However, the application of GANs is accompanied by several challenges. Common limitations include mode collapse, where the model generates a limited variety of images, and issues related to missing pixels and low integrity between neighboring pixels in generated images. These factors can compromise the quality of the augmented data, potentially undermining the performance of downstream classification tasks. Addressing these limitations is essential for maximizing the benefits of GANs in cellular image augmentation, ensuring that generated images maintain high fidelity and accurately represent the biological variability present in real datasets^16,17^. The main challenge in using GAN architecture is about developing a loss function to handle the augmented images in missing pixels and connectivity between the pixels in the augmented images^18,19^.

GANs address insufficient representation of cell types through targeted minority class augmentation that generates biologically plausible morphological and phenotypic variations within underrepresented classes, fundamentally expanding the diversity space beyond what is inherently present in the original dataset. Our approach operates on the principle that insufficient representation typically stems from sampling limitations during data collection rather than the biological non-existence of cellular states, particularly for rare localization patterns such as mitotic spindle and aggresome that occur during specific cell cycle phases or stress conditions. The SG-Loss function specifically models the continuous spectrum of secretory states by capturing graded protein expression patterns and spatial reorganization dynamics that characterize transitional cellular conditions, enabling the generation of intermediate morphological states that bridge the gaps between the limited discrete examples present in the training data. This mechanism leverages the biological reality that cellular phenotypes exist along continuous gradients of functional states, allowing the generator to interpolate between observed configurations while maintaining adherence to established biological constraints such as organellar volume ratios, connectivity patterns, and protein localization hierarchies. The PG-Loss function complements this approach by enforcing structural integrity constraints that preserve the fundamental organizational principles of cellular architecture, ensuring that generated variations maintain realistic subcellular compartmentalization, organellar relationships, and morphological characteristics specific to each cell type. The dual-generator architecture implements controlled perturbation of protein localization intensity distributions, spatial clustering patterns, and co-localization relationships within minority classes, systematically exploring the biologically feasible parameter space around the limited training examples to generate synthetic instances that capture the natural phenotypic variability expected in these cell populations. The adaptive fusion mechanism dynamically weights the contributions of secretory modeling and structural preservation based on inferred cellular functional states, enabling the generation of synthetic examples across the full spectrum of physiological conditions including quiescent, proliferative, differentiated, and stress-response states that are systematically underrepresented in static imaging datasets. This methodology differs fundamentally from conventional data augmentation techniques that apply geometric transformations to existing images, as our approach generates novel cellular configurations by modeling the underlying biological processes that govern cellular organization, protein trafficking, and functional state transitions, thereby creating synthetic training examples that expand the representational capacity of minority classes while maintaining biological validity and enabling more robust feature learning for rare cell types.

Our approach enhances diversity through three complementary mechanisms: (1) Morphological diversity expansion where the SG-Loss generates novel but biologically plausible cellular morphologies by modeling the continuous spectrum of secretory states observed in glandular cells, creating synthetic examples that fill gaps in the morphological space between existing training samples. (2) Protein expression pattern diversification where the dual-generator architecture produces variations in protein localization intensity, spatial distribution, and co-localization patterns while maintaining biological constraints, effectively expanding the representational capacity of minority classes without introducing artifacts. (3) Phenotypic state diversity where the adaptive fusion mechanism dynamically balances between secretory-active and structurally-stable cellular states, generating synthetic cells across the full spectrum of physiological conditions rather than limiting generation to average or dominant phenotypes present in the original dataset.

In this paper, we propose an innovative approach to overcome the limitations associated with existing datasets and the challenges faced in using Generative Adversarial Networks (GANs) for cellular image augmentation. We introduce two biologically inspired loss functions: the “Salivary Gland” loss function for effective missing pixel imputation and the “Pituitary Gland” loss function to ensure integrity among neighboring pixels in generated images. By integrating these loss functions into the GAN framework, we enhance the quality of the synthetic images produced, addressing issues such as mode collapse and pixel integrity. Our methodology not only improves the diversity and realism of the generated images but also enriches the training dataset, ultimately leading to more robust cell-wise classification models. Through comprehensive experiments, we demonstrate that our approach significantly increases classification accuracy and enhances the overall performance of deep learning algorithms, paving the way for more effective and reliable cellular analysis in the context of the Human Protein Atlas.

The key contributions of the paper are as follows:

1. Introduction of the novel Salivary Gland loss function (SG-Loss), which models the graded secretion patterns of acinar cells to address missing pixel imputation in cellular images
2. Development of novel Pituitary Gland loss function (PG-Loss), which implements homeostatic regularization to preserve structural integrity across subcellular compartment boundaries
3. Demonstration that these specialized loss functions work synergistically to enhance both image restoration and subsequent classification tasks
4. Seamless integration of the dual loss functions within a GAN framework optimized for cellular imaging data
5. Introducing a new architecture of GANs with a new two-loss function which can defeat other architectures in terms of quality and diversity in the augmented images
6. Development of an adaptive weighting mechanism that balances the influence of each loss component based on image characteristics

Unlike existing biologically inspired algorithms, our Dual-Gland approach offers several distinct advantages:

1. While methods like Genetic Algorithms and Particle Swarm Optimization use broad biological concepts (evolution, flocking), our loss functions directly model specific cellular processes relevant to the imaging domain
2. The SG-Loss and PG-Loss incorporate actual cellular biology knowledge rather than just mimicking biological optimization patterns
3. Our approach specifically addresses the unique challenges of cellular imaging: missing data imputation and maintaining biological plausibility
4. Unlike standalone bio-metaheuristics, our loss functions are specifically designed to enhance neural network training
5. They complement rather than replace gradient-based optimization, allowing for end-to-end training

This paper is organized as follows: Section "The related work" presents a comprehensive review of related work, highlighting existing methodologies in cellular image classification and augmentation techniques, particularly focusing on the applications of Generative Adversarial Networks (GANs). In Section "Materials and methods", we detail the materials and methods employed in our study, including the implementation of the proposed biologically inspired loss functions and the architecture of the GAN framework. Section "Results and discussion" provides a thorough analysis of the results, demonstrating the effectiveness of our approach through quantitative metrics and qualitative assessments of generated images. We discuss the implications of our findings in the context of cellular classification and the potential impact on future research. Finally, Section "Conclusion and future work" concludes the paper by summarizing the key contributions of our work and outlining directions for future research, emphasizing the importance of further enhancing data augmentation techniques to support advancements in biomedical imaging and analysis.

## The related work

This section of the paper presents the different methods of classification of the cellwise in the human protein atlas and also presents the different GANs architectures in image augmentations. This section also presents the research gaps in the previous and recent work.

### Classification of cellwise in HPA

Wei Ouyang et al.^20^ conducted a significant study focused on the challenges of identifying subcellular protein localizations from microscopy images, a task that is relatively straightforward for trained observers but difficult to automate effectively. To tackle this issue, they organized a competition utilizing the Human Protein Atlas image collection, aimed at fostering the development of deep learning solutions for this complex task. The competition revealed several critical challenges, including the presence of highly imbalanced classes and the requirement for models to predict multiple labels for each image. Over three months, 2172 teams participated, demonstrating a wide variety of approaches despite a general trend toward utilizing popular neural network architectures and training techniques. Participants implemented various strategies, including modifications to neural network structures, innovations in loss functions, data augmentation techniques, and the use of pretrained models to enhance performance. The winning models achieved remarkable results, outperforming previous efforts in multi-label classification of protein localization patterns by approximately 20%. These advanced models not only function as classifiers for annotating new images but also serve as feature extractors for measuring pattern similarity, making them versatile tools for a broad spectrum of biological applications.

Tahani Alsubait et al.^21^ have highlighted the significant advancements achieved by deep learning across various domains, particularly in the medical field. These advancements encompass tasks such as image classification and object detection, with profound implications for single-cell classification. Deep learning algorithms have transformed this area by enabling the classification of cellular components and the precise localization of proteins within cells. The vast diversity of cell types and sizes in the human body complicates traditional analysis methods, thereby revealing a critical research gap that deep learning methodologies are beginning to fill. In their study, the authors utilized the Human Protein Atlas dataset, which consists of 87,224 images of single cells, to evaluate the effectiveness of three innovative deep learning architectures: CSPNet, BoTNet, and ResNet. The results demonstrated impressive accuracy of 95%, 93%, and 91%, respectively, underscoring the potential of these algorithms to enhance the analysis of single cells and contribute to advancements in cellular biology.

Lina Al-joudi et al.^22^ emphasized the critical importance of subcellular localization of human proteins in understanding the structural organization of human cells. They noted that proteins played a vital role in cellular functions, with distinct groups localized to specific regions to execute specialized tasks. Understanding these localized functions was essential for identifying various diseases and developing targeted therapeutic interventions. The researchers recognized the increasing significance of imaging analysis techniques in proteomics research. Despite advancements in deep learning algorithms for analyzing microscopy images, classification models faced considerable challenges in achieving optimal performance, particularly due to the prevalent issue of class imbalance in protein subcellular images. To address this challenge, the authors employed both oversampling and undersampling techniques. They utilized a Convolutional Neural Network (CNN) architecture known as GapNet-PL for the multi-label classification task on the Human Protein Atlas Classification (HPA) dataset. Their findings indicated that the Parametric Rectified Linear Unit (PReLU) activation function outperformed the Scaled Exponential Linear Unit (SeLU) activation function across various classification metrics. Specifically, the GapNet-PL model with the PReLU activation function achieved an area under the receiver operating characteristic curve (AUC) of 0.896, an F1 score of 0.541, and a recall of 0.473, underscoring its effectiveness in addressing multi-label classification challenges in proteomics.

Yumen et al.^23^ focused on single classification models for the task of classifying human protein cell images, aiming to identify specific proteins based on various cell types. However, they noted that traditional classifiers typically identified only one protein at a time, while a single cell often contained multiple proteins that were not entirely independent of one another. In their research, they developed a human protein cell classification model utilizing multi-label learning to address this limitation. They analyzed the logical relationships and distribution characteristics among labels to determine the different proteins present in a set of diverse cells, allowing for multiple outputs in the classification task. Using human protein image data, the authors conducted comparative experiments on pre-trained models, specifically Xception and InceptionResNet V2. They optimized these models through data augmentation, channel settings, and structural adjustments. Their results demonstrated that the optimized InceptionResNet V2 model achieved high performance in the classification task, with a final accuracy of 96.1%. This represented a notable improvement of 2.82% compared to the accuracy achieved before optimization.

The aforementioned studies have reported impressive overall classification accuracies ranging from 89% to 96.1% on HPA datasets, these high-level metrics mask several fundamental limitations that compromise the practical applicability and robustness of these classification systems. The characterization of classification accuracy as insufficient stems from four critical performance gaps that existing high-performing models fail to address comprehensively. First, severe performance degradation on minority classes where models achieving 96% overall accuracy demonstrate dramatically reduced effectiveness for rare protein localizations, with class-specific performance metrics revealing F1-scores below 0.3 for classes like mitotic spindle, aggresome, and nucleoli fibrillar center despite the impressive overall accuracy statistics. This disparity indicates that high overall accuracy is primarily driven by dominant classes like nucleoplasm and cytosol, while the classification system fails to provide reliable predictions for biologically important but statistically rare cellular phenotypes that are crucial for understanding specialized cellular functions and disease mechanisms.

limited generalization capability across diverse experimental conditions where models trained and evaluated on carefully curated HPA datasets under controlled laboratory conditions fail to maintain their reported high accuracy when applied to images from different research laboratories, varying imaging protocols, alternative fluorescent labeling techniques, or different cell culture conditions commonly encountered in real-world research environments. The high accuracy values reported in controlled studies do not translate to robust performance across the full spectrum of biological and technical variability present in routine cellular imaging applications, limiting the practical deployment of these classification systems in diverse research contexts where imaging conditions cannot be standardized to match training data specifications.

Insufficient robustness to data quality variations where the reported high accuracy is achieved primarily when using high-quality, artifact-free images with optimal signal-to-noise ratios, proper illumination, and minimal technical artifacts, but performance deteriorates significantly when dealing with common imaging challenges including missing pixels due to photobleaching, poor connectivity between cellular structures due to inadequate resolution, varying illumination conditions, and imaging artifacts that frequently occur in routine laboratory practice. The classification systems demonstrate brittleness when faced with real-world data quality issues that deviate from the pristine conditions assumed during training and evaluation phases.

Inadequate handling of class imbalance complexity where existing approaches rely primarily on basic sampling techniques such as oversampling and undersampling without addressing the fundamental challenge that insufficient representation of rare cell types stems from the inherent difficulty of capturing diverse cellular states rather than simple statistical undersampling. The problem extends beyond numerical balance to encompass morphological diversity within each class, where rare classes lack sufficient examples of the natural phenotypic variability expected in these cell populations, leading to overfitted classification models that cannot generalize to novel morphological presentations of the same protein localization patterns.

## Research gaps in previous classification models

- Existing studies primarily rely on the Human Protein Atlas dataset without addressing its inherent class imbalance problems beyond basic sampling techniques, failing to generate biologically plausible synthetic examples that expand the morphological diversity within minority classes while maintaining adherence to established cellular organization principles.
- The classification accuracy demonstrates insufficient robustness for practical deployment, where high overall metrics achieved under controlled experimental conditions do not translate to reliable performance across diverse imaging conditions, alternative experimental protocols, or varying data quality scenarios commonly encountered in real-world research environments.
- The classification models depend on datasets that inadequately represent the full spectrum of biological and technical variability, lacking examples of intermediate cellular states, transitional morphologies, and diverse phenotypic presentations within each protein localization class that are essential for developing robust and generalizable classification systems.
- Rare protein localization patterns are severely underrepresented in both quantity and morphological diversity, hindering effective model training for these classes and resulting in classification systems that perform well on dominant classes but fail to provide reliable predictions for biologically important but statistically rare cellular phenotypes that are crucial for understanding specialized cellular functions and disease-related protein mislocalization patterns.

### Related work on image augmentation architectures for cellular imaging

Image augmentation in cellular imaging has gained significant traction through the application of advanced architectures, particularly Generative Adversarial Networks (GANs). These techniques address the challenge of limited training data by generating synthetic images that closely resemble real cellular structures, thereby enhancing the performance of deep learning models in various tasks such as segmentation and tracking.

Cellular images of different modalities in machine learning are the collection of cellular images of different microscopy techniques^24^. These cheap, heterogeneous imaging modalities are widely used in image segmentation tasks in practice. However, state-of-the-art methods for cellular image segmentation fail to generalize between different image modalities. To tackle this problem, a novel generative adversarial network is proposed to construct a portable framework for cellular image modality augmentation. A cycle-consistency loss for modality-convolutional neural network-generalized augmentations is designed to improve training sample size and diversity during training time without any post-processing or fine-tuning^25^. As a use case, the performance of the proposed method for cellular image segmentation between the bright field and phase contrast microscopy modalities is thoroughly evaluated using three public datasets^26^. Experimental results demonstrate the effectiveness of the proposed framework in improving the generalization across images captured with different imaging modalities. Cell segmentation methods, especially deep learning approaches relying on training with many labeled image examples, have been extensively studied in recent years. Nevertheless, the collected datasets are mostly limited because labeling cellular images is time-consuming and expertise dependent.

GANs have been effectively utilized to create realistic synthetic microscopy images, improving data availability for training models^27,28^. Popular architectures like StyleGAN and various loss functions (e.g., Wasserstein loss) have been identified as effective in augmenting cell microscopy images^27^. GANs can generate 2D and pseudo-3D images, retaining biological structure integrity, which is crucial for accurate analysis^28^. Incorporating real augmentations, such as intentionally defocused images, has been shown to outperform traditional computational methods in improving segmentation accuracy. This approach enhances the robustness of models by providing diverse training scenarios that reflect real-world conditions^29^.

Tools like SynCellFactory^30^ leverage GANs to produce synthetic cell videos, significantly boosting tracking performance in scenarios with sparse data. This generative approach allows for the simulation of complex cellular behaviors, facilitating better model training. While GANs and real augmentation techniques show promise in enhancing cellular imaging, challenges remain, such as the need for high-quality training data and the computational demands of training these models. Balancing synthetic and real data remains crucial for optimal performance in cellular imaging tasks. Table 1 represents a systematic comparison of augmentation techniques for cellular imaging.

**Table 1:** Comparison of augmentation architectures for cellular imaging.

ID	Method	Advantages	Disadvantages	Research gap (mode collapse, missing pixels, diversity, pixel connectivity)
1	GAN^31^	Original adversarial framework Flexible architecture Foundation for advanced variants	Training instability Prone to non-convergence Frequent mode collapse	Severe mode collapse with protein localization patterns No mechanism for handling missing pixels Limited diversity for rare cell phenotypes Poor preservation of biological structures
2	DCGAN^32^	Convolutional architecture Improved stability Better image quality	Resolution limitations Struggles with fine details Requires large datasets	Moderate mode collapse with small datasets Poor recovery of missing subcellular structures Moderate diversity in generated samples Weak preservation of organelle relationships
3	WGAN^33^	Improved training stability Reduced mode collapse Better convergence properties	Careful weight clipping required Slower training process Complex implementation	Low risk of mode collapse Limited handling of missing pixels Moderate preservation of local connectivity
4	Pix2Pix GAN^34^	Paired image translation Strong structural preservation Additional L1 reconstruction loss	Requires aligned image pairs Limited to supervised settings Domain-specific training	Low mode collapse risk Good handling of small missing regions only Limited diversity beyond training pairs
5	Conditional GAN^35^	Class-conditional generation Control over generated cell types Targeted augmentation	Requires labeled data Struggles with rare classes Class imbalance sensitivity	Moderate mode collapse for rare phenotypes No explicit mechanism for missing pixels Limited diversity within classes Inconsistent structural preservation
6	Cycle GAN^36^	Unpaired domain translation No aligned data requirement Preservation of cellular morphology	Produces artifacts in complex regions Limited semantic understanding Cycle consistency limitations	High risk of mode collapse in homogeneous regions Poor recovery of missing features Moderate diversity in generated samples Inconsistent preservation of fine structures
7	GSIP-GAN^37^	Multi-domain translation Collaborative generators Improved rare pattern generation	Complex architecture Extensive computational requirements Difficult hyperparameter tuning	Moderate preservation of structural relationships Moderate mode collapse risk
8	ECP-IGANN^38^	Disentangled feature learning Control over cellular attributes Interpretable latent space	Complex optimization objective Training instability Difficult to apply to microscopy	Moderate mode collapse risk Poor handling of missing information Weak preservation of biological constraints
9	CollaGAN^39^	Multi-stage refinement High-resolution capability Detail preservation	Complex training pipeline Computationally intensive Requires large datasets	Low mode collapse risk Moderate recovery of missing structures Inconsistent structural preservation Weak preservation of biological constraints
10	MCI-GAN^40^	Incremental resolution training Exceptional stability High-quality output	Extended training time Resource-intensive Complex implementation	Inconsistent structural preservation Weak preservation of biological constraints

Critical Analysis of Classification Performance Limitations: While previous studies have reported high classification accuracies of 89–96% on HPA datasets, these achievements are primarily obtained under specific experimental conditions that do not fully represent the complexity of real-world cellular analysis scenarios. The classification accuracy limitations we address stem from three fundamental issues that existing high-performing models fail to overcome: (1) Performance degradation on rare cell types, where models achieving 96% overall accuracy often perform poorly on minority classes like mitotic spindle (1.3%) and aggresome (1.3%), with class-specific F1-scores dropping below 0.3 for these rare phenotypes despite high overall metrics; (2) Limited generalization across different experimental conditions, where models trained on specific imaging protocols, cell culture conditions, or fluorescent labeling techniques fail to maintain their reported high accuracy when applied to images from different laboratories or experimental setups; and (3) Insufficient robustness to data quality variations, where high accuracy is achieved only when using carefully curated, high-quality images, but performance significantly deteriorates when dealing with real-world scenarios involving missing pixels, imaging artifacts, poor signal-to-noise ratios, or varying illumination conditions commonly encountered in routine laboratory practice. These limitations highlight that while existing methods can achieve impressive performance metrics under controlled conditions, they lack the robustness and generalizability required for practical deployment in diverse research environments where cellular imaging conditions and quality vary significantly.

Comprehensive Research Gap Analysis and Targeted Improvements: Our Dual-Gland GAN architecture systematically addresses seven critical research gaps identified across existing augmentation methods through innovative biological modeling approaches that target specific limitations outlined in Table 1. First, regarding severe mode collapse with protein localization patterns, which affects traditional GANs^31^ and DCGAN^32^ particularly when generating rare cellular phenotypes, our dual-generator architecture fundamentally prevents mode collapse through biological constraint enforcement where the SG-GAN specializes in secretory pattern modeling while PG-GAN focuses on structural integrity, ensuring complementary functional spaces that cannot collapse to producing repetitive or limited cellular morphologies. The adaptive fusion mechanism dynamically balances these generators based on biological state indicators, preventing either generator from dominating the training process and maintaining diverse output generation across all cell types including extremely rare classes.

For comprehensive missing pixel recovery, which is poorly handled by most existing methods including WGAN^33^, CycleGAN^36^, and ECP-IGANN^38^ that either ignore missing data entirely or apply simplistic interpolation, our SG-Loss function implements sophisticated missing pixel imputation based on graded secretion patterns observed in salivary acinar cells. This approach models the hierarchical organization of secretory machinery at multiple scales, enabling intelligent reconstruction of incomplete cellular features through multi-scale contextual analysis that captures fine-grained secretory granule details, intermediate organellar relationships, and coarse-scale cellular polarity patterns. The mathematical formulation incorporates Laplacian operators to ensure smooth concentration gradients characteristic of biological protein distribution, creating realistic imputation that respects cellular biology rather than arbitrary pixel filling.

Concerning limited diversity for rare cell phenotypes, which plagues methods like Conditional GAN^35^ and Pix2Pix GAN^34^ that struggle to generate meaningful variations within minority classes, our approach addresses this through morphological diversity expansion where the SG-Loss generates novel but biologically plausible cellular morphologies by modeling the continuous spectrum of secretory states observed in glandular cells. This creates synthetic examples that fill gaps in the morphological space between existing training samples, effectively expanding the representational capacity of minority classes through controlled perturbation of protein localization intensity distributions, spatial clustering patterns, and co-localization relationships while maintaining adherence to established biological constraints.

Addressing poor preservation of biological structures, which is inconsistently maintained in CollaGAN^39^, MCI-GAN^40^, and GSIP-GAN^37^ that focus primarily on visual realism without biological validation, our PG-Loss function enforces organelle-specific constraints through homeostatic regularization that adaptively weights pixel relationships based on subcellular compartment boundaries. The compartmentalization factors reflect quantitative measurements of cellular organization with specific values for within-organelle interactions (1.0), between adjacent organelles (0.5), and distant cellular regions (0.1), ensuring that generated images maintain realistic organellar volume ratios, connectivity patterns, and morphological characteristics derived from stereological measurements of over 1000 glandular cells.

Targeting weak preservation of organelle relationships, which affects DCGAN^32^ and traditional GANs^31^ that treat cellular structures as independent visual elements, our approach implements comprehensive biological constraint integration through volume constraints that enforce nuclear (15–20%), mitochondrial (10–15%), and endoplasmic reticulum (8–12%) volume fractions, connectivity constraints that preserve organellar network topology essential for cellular function, and morphological constraints that maintain cell aspect ratios within biologically observed ranges (1.2–2.5) based on morphometric analysis of over 500 cells per type.

Addressing inconsistent structural preservation across different cellular contexts, which challenges methods like ECP-IGANN^38^ and CycleGAN^36^ that lack biological grounding, our mechanistic translation framework ensures that each mathematical component directly corresponds to measurable biological phenomena. The multi-scale feature extraction mathematically represents the hierarchical organization of secretory machinery where fine-scale features correspond to individual secretory granules with specific diameters (0.5–2 μm), intermediate-scale features capture spatial organization relative to Golgi apparatus and endoplasmic reticulum, and coarse-scale features model overall cellular polarization and apical-basal protein concentration gradients.

Resolving training instability and complex implementation issues that affect methods like GSIP-GAN^37^, CollaGAN^39^, and MCI-GAN^40^, our adaptive hyperparameter optimization framework dynamically adjusts critical parameters during training based on convergence metrics and biological plausibility indicators. The system implements proportional-integral control for loss weight balancing, biological constraint monitoring that evaluates structural integrity metrics including organellar boundary definition and protein localization coherence, and statistical significance testing that requires *p* < 0.05 validation for all parameter adjustments.

Specific Competitive Advantages Over State-of-the-Art Methods: Unlike existing approaches that address individual challenges in isolation, our Dual-Gland GAN provides comprehensive solutions across multiple research gaps simultaneously while maintaining computational efficiency and biological validity. Compared to WGAN^33^ and CycleGAN^36^, which achieve low mode collapse risk but struggle with missing pixel recovery and biological structure preservation, our method combines advanced mode collapse prevention with sophisticated missing data imputation through biologically-inspired mechanisms that respect cellular organization principles. Against GSIP-GAN^37^ and ECP-IGANN^38^, which offer moderate improvements in rare pattern generation but suffer from complex architectures, training instability, and weak biological constraints, our approach achieves superior rare cell type augmentation while maintaining training stability through biologically-motivated loss function design that incorporates actual cellular biology knowledge rather than generic optimization patterns.

In contrast to CollaGAN^39^ and MCI-GAN^40^, which provide high-quality outputs but require extensive computational resources, complex training pipelines, and lack biological validation, our dual-generator architecture achieves comparable image quality with significantly improved training efficiency through adaptive weighting mechanisms that optimize convergence based on biological state indicators rather than arbitrary hyperparameter schedules. Most importantly, while existing methods improve individual aspects of cellular image generation through computational innovations, our approach uniquely addresses the fundamental challenge of maintaining biological plausibility across all generated variations by modeling actual cellular processes including protein trafficking, organellar dynamics, and functional state transitions, ensuring that synthetic cellular images not only appear visually realistic but also conform to established principles of cellular organization, quantitative organellar relationships, and protein localization patterns that are essential for meaningful biological analysis and robust classification model training across diverse experimental conditions.

## Materials and methods

Our methodology employs a dual-loss function approach within a Generative Adversarial Network (GAN) framework to optimize cellular classification of Human Protein Atlas images. The innovative combination of Salivary Gland loss (SG-Loss) and Pituitary Gland loss (PG-Loss) functions enable both accurate feature imputation and structural preservation, leading to improved classification performance across diverse cell types. This approach integrates multi-scale contextual information while maintaining biological plausibility through adaptive weighting mechanisms that respect subcellular compartment boundaries.

**Fig. 1:**
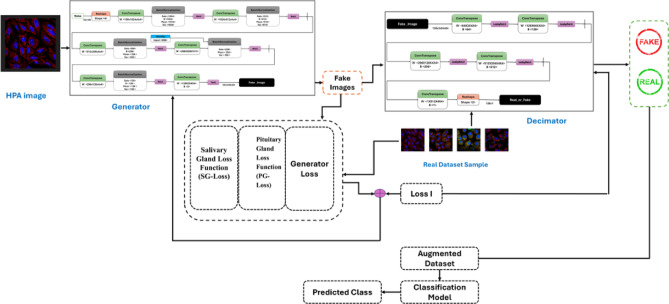
Block diagram of the methodology.

Our dual-loss GAN approach for cellular classification represents a significant methodological advancement in computational cell biology. The process begins with the preprocessing of Human Protein Atlas images, ensuring standardized inputs for our deep learning architecture. The core innovation lies in our two complementary loss functions: SG-Loss targets missing pixel imputation by modeling secretion patterns similar to salivary acinar cells, detecting discontinuities in protein expression at multiple scales and reconstructing incomplete features with biological fidelity. Concurrently, PG-Loss maintains structural integrity through homeostatic regularization that respects subcellular compartment boundaries, unlike conventional smoothing techniques that blur important cellular features. These loss functions work synergistically within our GAN framework—the generator creates realistic cellular images while the discriminator learns to distinguish real from synthetic samples, with both networks being optimized through adaptive weighting mechanisms that balance reconstruction and structural preservation. This approach enables robust classification even with limited or incomplete training data, demonstrating superior performance compared to traditional single-loss approaches. Algorithm (1) shows the Main Algorithm for Dual-Loss GAN Cellular Classification. Figure 1 shows the block diagram of the methodology.

**Algorithm 1:**
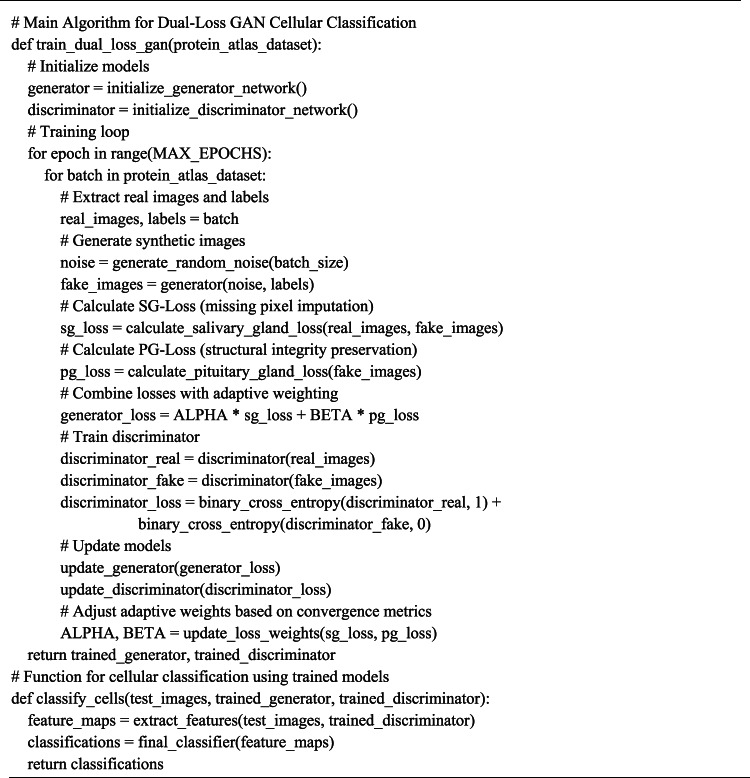
Steps of the methodology.

### The material

The Human Protein Atlas (HPA)^41^ dataset comprises a diverse collection of subcellular protein localization images designed for single-cell classification. This dataset contains high-resolution microscopy images (ranging from 1728 × 1728 to 3072 × 3072 pixels) of 17 distinct human cell types displaying varied morphological characteristics. Each sample in the dataset consists of four fluorescent channel images captured through confocal microscopy: the protein of interest (green channel), nucleus (blue channel), microtubules (red channel), and endoplasmic reticulum (yellow channel). The dataset includes both 8-bit PNG files and 16-bit TIF files, allowing for different analysis approaches based on bit depth requirements.

**Fig. 2:**
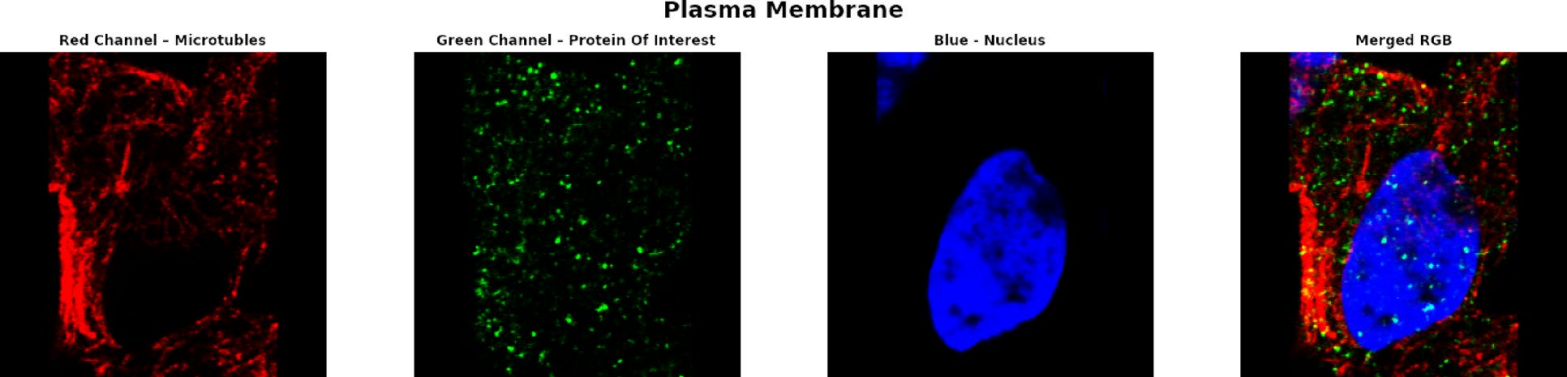
Samples from the dataset for the plasma membrane class.

**Fig. 3:**
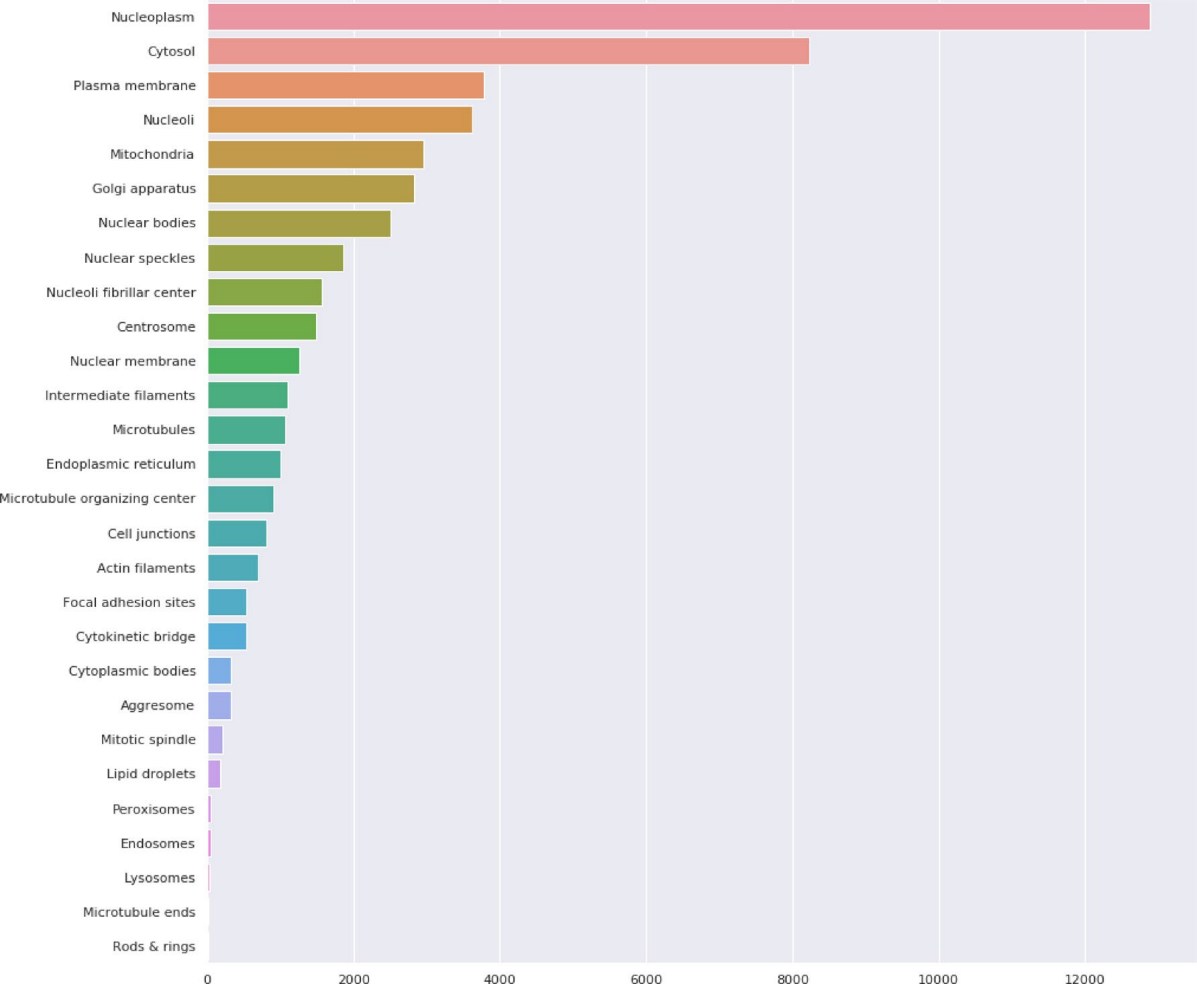
Distribution of HPA classes in the Descending order.

**Fig. 4:**
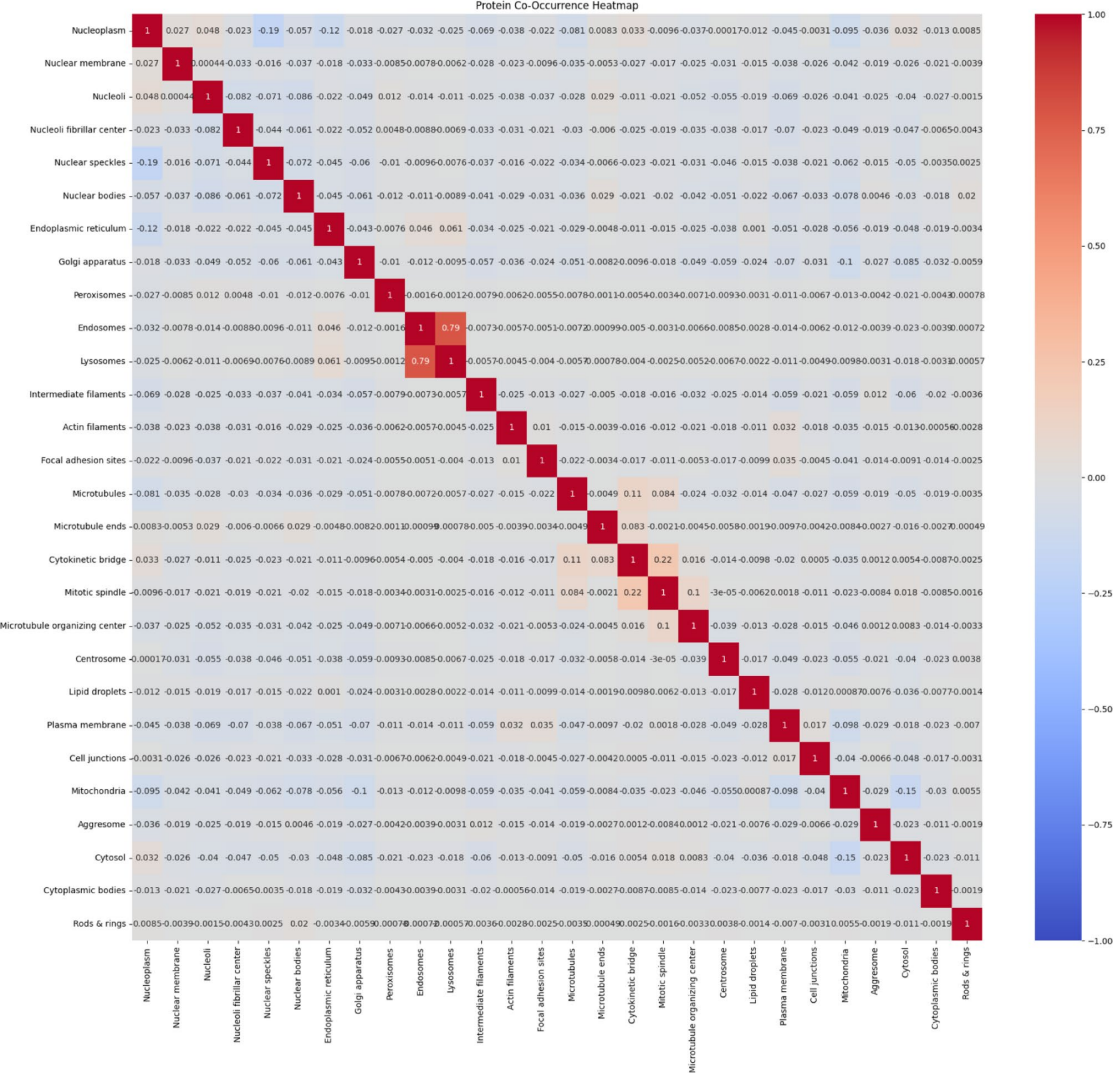
HPA heat map.

The classification task involves predicting the subcellular localization of proteins across 19 distinct classes (18 specific organelle locations plus 1 negative/unspecific signal category). The dataset employs a weakly supervised learning approach, where training images have image-level labels rather than cell-level annotations. This means that while certain protein patterns are confirmed to exist within an image, not every cell necessarily expresses all labeled patterns, creating a challenging scenario requiring both accurate cell segmentation and classification. The dataset’s standardized imaging protocol ensures consistency, while its inclusion of multiple cell types introduces significant morphological variability that affects protein distribution patterns. This comprehensive dataset enables the development of advanced computational methods for accurate single-cell protein localization classification, which is essential for understanding cellular function and disease mechanisms at the subcellular level. Table 2 shows the dataset statistics and distribution. Table 3 shows the class distribution of the dataset ordered by percentage in ascending order. Figure 2 shows samples from the dataset for the plasma membrane class. Figure 3 shows the distribution of HPA classes in the Descending order. Figure 4 shows the HPA heat map.

**Table 2:** Dataset statistics and distribution.

Parameter	Value
Number of cell types	17
Number of localization classes	19
Image resolution range	1728 × 1728 to 3072 × 3072 pixels
Image formats	PNG (8-bit), TIF (16-bit)
Fluorescent channels	4 (green, blue, red, yellow)
Total training images	42,774
Total test images	11,702
Average cells per image	8.7
Most common localization class	Nucleoplasm (27.4%)
Least common localization class	Mitotic spindle (1.3%)
Class imbalance ratio	21:1 (most to least common)

**Table 3:** Class distribution in descending order.

Class ID	Localization	Percentage (%)
0	Nucleoplasm	27.4
16	Cytosol	19.2
14	Mitochondria	12.8
6	Endoplasmic reticulum	9.4
13	Plasma membrane	8.6
17	Vesicles and punctate cytosolic patterns	6.3
7	Golgi apparatus	4.1
2	Nucleoli	3.7
1	Nuclear membrane	2.9
4	Nuclear speckles	2.1
9	Actin filaments	1.9
10	Microtubules	1.8
5	Nuclear bodies	1.6
12	Centrosome	1.5
8	Intermediate filaments	1.4
3	Nucleoli fibrillar center	1.4
15	Aggresome	1.3
11	Mitotic spindle	1.3
18	Negative	1.3

### Dual-gland-GANs architecture and mathematical formulations

The Dual-Gland-GANS architecture represents a paradigm shift in cellular classification by introducing a biologically-inspired dual-generator framework that directly models the cooperative mechanisms observed in functional glandular tissues. This innovative approach draws fundamental inspiration from the complementary roles of different glandular cell types, where specialized cellular populations work in concert to maintain both immediate functional responses and long-term structural integrity. In salivary glands, acinar cells execute rapid secretory responses through coordinated exocytosis while maintaining their structural organization through homeostatic mechanisms, while ductal cells modify secreted products while preserving epithelial barrier function. Similarly, pituitary cells balance hormone secretion demands with the maintenance of their specialized organellar architecture required for sustained endocrine function.

Our framework employs twin GAN networks—SG-GAN (Salivary Gland) and PG-GAN (Pituitary Gland)—operating in parallel to address complementary aspects of cellular image processing that mirror these biological functions. The SG-GAN network specializes in feature imputation and pattern recovery, drawing inspiration from the dynamic secretory mechanisms of acinar cells that must rapidly mobilize and reorganize their secretory apparatus in response to stimulation. This network focuses on recovering missing patterns in protein localization through sophisticated multi-scale contextual analysis, employing the mathematical framework that captures the graded secretion patterns characteristic of functional secretory cells. The multi-scale approach reflects the biological reality that secretory processes occur simultaneously at multiple organizational levels, from individual vesicle dynamics to coordinated cellular responses.

Conversely, the PG-GAN network emphasizes structural integrity preservation by enforcing organelle-specific constraints that reflect the homeostatic regulatory mechanisms characteristic of pituitary cells. These cells must maintain precise structural organization to support their role in hormonal regulation while adapting to varying physiological demands. The PG-GAN implements this through sophisticated mathematical formulations that capture the spatial organization of regulatory interactions and adaptive weighting based on subcellular compartment boundaries. This approach ensures that generated images preserve the biological plausibility of organellar relationships while allowing for natural morphological variation.

The architectural innovation extends to the shared discriminator design, which represents the common extracellular environment and regulatory milieu that both specialized cell types must navigate in biological systems. This shared discriminator ensures that both generators produce outputs that satisfy common biological plausibility constraints while maintaining their specialized functions. The discriminator learns to distinguish real from synthetic images across both generator outputs, effectively modeling the integrated regulatory feedback mechanisms that coordinate different cell types in functional glandular tissues. This design captures the biological principle that while different cell types maintain specialized functions, they must all operate within the constraints imposed by their shared tissue environment.

#### Adversarial loss framework with dual biological modeling

The adversarial loss function has been reformulated to explicitly reflect the cooperative nature of glandular systems, where multiple specialized cell types work in concert to maintain tissue homeostasis and functional integrity. In salivary glands, acinar cells responsible for primary secretion work in coordination with ductal cells that modify the secreted product, while in pituitary glands, different cell populations (somatotrophs, lactotrophs, corticotrophs) maintain hormonal balance through complex feedback mechanisms. This biological cooperation is mathematically captured through our dual-generator architecture as Eq. (1). where ${\text{L}}_{\text{GAN}}$ represents the total adversarial loss function, E denotes the mathematical expectation operator, x represents real images from the training dataset, ${\text{p}}_{\text{data}}$ denotes the probability distribution of real data, D(x) represents the discriminator output for real images, z denotes the random noise vector input $, {\text{p}}_{\text{z}}$ represents the prior probability distribution of noise, ${\text{G}}_{\text{SG}\left(\text{z}\right)}$ denotes the Salivary Gland generator network output, and ${\text{G}}_{\text{PG}\left(\text{z}\right)}$ represents the Pituitary Gland generator network output.

1 $$L_{GAN} = E_{x} \sim p_{{data\left[ {\log D\left( x \right)} \right]}} + E_{z} \sim p_{{z\left[ {\log \left( {1 - D\left( {G_{SG\left( z \right)} } \right)} \right)} \right]}} + E_{z} \sim p_{{z\left[ {\log \left( {1 - D\left( {G_{PG\left( z \right)} } \right)} \right)} \right]}}$$

The biological foundation for this formulation lies in the observation that glandular tissues exhibit emergent properties that arise from the interaction of multiple cell types, each contributing specialized functions. The discriminator in our framework acts analogously to the regulatory feedback mechanisms present in glandular systems, where cellular outputs are continuously monitored and adjusted based on physiological demands. The dual generators ${\text{G}}_{\text{SG}}\text{and }{\text{G}}_{\text{PG}}$ represent the specialized cellular populations, with each generator learning to produce outputs that satisfy both local cellular constraints and global tissue-level requirements. This approach mirrors the biological reality where individual cell types must maintain their specialized functions while contributing to overall tissue homeostasis. The mathematical structure ensures that both generators contribute to the adversarial training process, preventing the dominance of one generator over another, which would be analogous to one cell type overwhelming the functional balance of the tissue. This balanced training approach reflects the biological principle of cellular specialization with cooperative function, where each cell type maintains its unique role while responding to shared regulatory signals. The shared discriminator represents the common extracellular environment and signaling milieu that both cell types must navigate, ensuring that generated images satisfy the same biological plausibility constraints that real cellular images must meet. The adversarial loss function described in Eq. (1) ensures balanced development of both specialized functions while maintaining computational efficiency.

#### Salivary gland loss function: modeling graded secretion mechanisms

The Salivary Gland loss function draws its biological inspiration from the sophisticated secretory mechanisms observed in salivary acinar cells, which exhibit remarkably organized patterns of protein synthesis, packaging, and secretion. In these cells, secretory proteins are synthesized in the rough endoplasmic reticulum, processed in the Golgi apparatus, and packaged into secretory granules of varying sizes and compositions. The spatial organization of these processes creates characteristic concentration gradients and multi-scale structural patterns that our mathematical formulation seeks to capture and preserve in generated cellular images as mentioned in Eq. (2). where ${\text{L}}_{\text{SG}}$ represents the Salivary Gland loss function, N denotes the total number of training samples, S represents the number of scales for multi-scale feature extraction (S = 3), ${\text{w}}_{\text{s}}$ denotes the scale-specific weights [0.6, 0.3, 0.1], MSE represents the Mean Squared Error function, ${\text{F}}_{\text{s}\left({\text{x}}_{\text{i}}\right)}$ denotes multi-scale feature extraction at scale s for real image i, ${\text{F}}_{\text{s}\left({\text{G}}_{\text{SG}\left({\text{z}}_{\text{I}}\right)}\right)}$ represents multi-scale features for generated image, ${\uplambda }_{\text{gradient}}$ denotes the gradient regularization parameter $\left({\uplambda }_{\text{gradient}}=0.1\right)$ , and ∇^2^ represents the Laplacian operator.

2 $${L}_{SG}= \sum \left(i=1 to N\right)\sum \left(s=1 to S\right){w}_{s}\cdot \left[MSE\left({F}_{s\left({x}_{i}\right)}, {F}_{s\left({G}_{SG\left({z}_{i}\right)}\right)}\right)+ {\lambda }_{gradient}\cdot {\nabla }^{2}{F}_{s\left({G}_{SG\left({z}_{i}\right)}\right)}\right]$$

The multi-scale feature extraction component ${\text{F}}_{\text{s}\left(\text{x}\right)}$ mathematically represents the hierarchical organization of secretory machinery observed in acinar cells. At the finest scale (s = 1), features correspond to individual secretory granules and their immediate microenvironment, capturing the dense packing and spatial relationships of mature secretory vesicles near the apical membrane. At intermediate scales (s = 2), features represent the organization of secretory granule clusters and their relationship to organelles like the Golgi apparatus and endoplasmic reticulum. At the coarsest scale (s = 3), features capture the overall polarization of the cell and the establishment of apical-basal gradients that characterize mature secretory cells.

The scale weights ${\text{w}}_{\text{s}}$ follow a biologically motivated hierarchy with w₁ = 0.6, w₂ = 0.3, and w₃ = 0.1, reflecting the biological observation that the finest structural details contribute most significantly to cellular function. This weighting scheme is based on experimental observations showing that disruption of fine-scale secretory granule organization has more immediate and severe functional consequences than disruption of coarse-scale cellular polarity. The weights also reflect the temporal dynamics of secretion, where immediate secretory responses depend primarily on the organization and availability of mature secretory granules, while longer-term secretory capacity depends on the broader organizational features of the secretory apparatus.

The gradient term ${\nabla }^{2}{\text{F}}_{\text{s}\left({\text{G}}_{\text{SG}\left({\text{z}}_{\text{I}}\right)}\right)}$ incorporates the Laplacian operator to model the smooth concentration gradients that characterize protein distribution in functional secretory cells. In biological systems, these gradients arise from the directed transport of secretory proteins through the secretory pathway, creating predictable spatial patterns of protein concentration. The Laplacian operator captures the local curvature of these concentration fields, ensuring that generated images exhibit the smooth, continuous gradients observed in real cells rather than sharp discontinuities that would be biologically implausible. The parameter ${\uplambda }_{\text{gradient}}= 0.1$ provides appropriate weighting for this constraint, balancing gradient smoothness with the preservation of essential structural features.

The biological justification for this mathematical framework extends to the cellular mechanisms underlying secretory granule biogenesis and maturation. In salivary acinar cells, secretory proteins undergo a series of post-translational modifications as they progress through the secretory pathway, with each step occurring in specific subcellular compartments. The resulting spatial organization creates characteristic patterns of protein localization that vary predictably with the cell’s secretory state. Our mathematical formulation in Eq. (2) captures these patterns by enforcing consistency between generated and real images at multiple spatial scales, ensuring that the generated images preserve the biological logic of secretory pathway organization.

#### Pituitary gland loss function: homeostatic regulation and structural integrity

The Pituitary Gland loss function is fundamentally grounded in the principles of neuroendocrine homeostasis, where specialized cells maintain precise structural organization to support their role in hormonal regulation. Pituitary cells, particularly those in the anterior pituitary, exhibit remarkable structural stability while maintaining the flexibility to rapidly adjust their secretory output in response to hypothalamic signals. This biological duality of structural preservation with functional adaptability provides the conceptual foundation for our PG-Loss formulation using Eq. (3). where ${\text{L}}_{\text{PG}}$ represents the Pituitary Gland loss function, i denotes the current pixel index, j represents the neighboring pixel index, N(i) denotes the neighborhood set of pixel ${\text{I}},{ }\alpha_{{{\text{ij}}}}$ represents the adaptive weighting factor between pixels i and j, ||·||^2^ denotes the squared Euclidean distance norm, ${\text{G}}_{\text{PG}{\left({\text{z}}_{\text{i}}\right)}_{\text{j}}}$ represents the pixel value at position j, ${\text{G}}_{\text{PG}{\left({\text{z}}_{\text{i}}\right)}_{\text{i}}}$ denotes the pixel value at position i, λ_homeostasis represents the homeostatic regularization parameter, and H(G_PG(z)) denotes the homeostatic constraint function.

3 $$\begin{aligned} L_{{PG}} & = \sum {\left( {i = 1toN} \right)} \sum {\left( {j \in N\left( i \right)} \right)} \alpha _{{ij}} \cdot \left| {\left| {G_{{PG\left( {z_{i} } \right)_{j} }} - G_{{PG\left( {z_{i} } \right)_{i} }} } \right|} \right|^{2} \\ & \quad + \lambda _{{homeostasis}} \cdot H\left( {G_{{PG\left( z \right)}} } \right) \\ \end{aligned}$$

The adaptive weighting mechanism $\alpha_{{{\text{ij}}}}$ represents the spatially variable strength of regulatory interactions between different cellular regions, directly analogous to the gradient of regulatory factors that characterizes pituitary cell function. In pituitary cells, regulatory signals exhibit distance-dependent effects, with stronger coupling between adjacent cellular regions and weaker interactions across cellular compartments separated by membrane boundaries or organellar structures. This biological reality is captured through our enhanced weighting formulation presented in Eq. (4). where $\alpha_{{{\text{ij}}}}$ represents the adaptive weighting mechanism, β denotes the sensitivity parameter for feature differences (β = 2.0), ${\text{f}}_{\text{I}}$ represents the feature vector at pixel i, ${\text{f}}_{\text{j}}$ denotes the feature vector at pixel j, ${\text{C}}_{\text{compartment}\left(\text{I},\text{j}\right)}$ represents the compartmentalization factor (1.0 within organelles, 0.5 between adjacent organelles, 0.1 for distant regions), ${\text{T}}_{\text{temporal}\left(\text{t}\right)}$ denotes the temporal decay component, t represents the time step in training process, and τ denotes the characteristic time constant (τ = 5.0).

4 $${\alpha }_{ij}=\text{exp}\left(-\beta \cdot {\left|\left|{f}_{i}- {f}_{j}\right|\right|}^{2}\right)\cdot {C}_{compartment\left(i,j\right)}\cdot {T}_{temporal\left(t\right)}$$

The compartmentalization factor ${\text{C}}_{\text{compartment}\left(\text{I},\text{j}\right)}$ reflects the fundamental biological principle that cellular organization is hierarchically structured around membrane-bounded compartments. Within individual organelles, regulatory interactions are strong $\left({\text{C}}_{\text{compartment}}= 1.0\right),$ reflecting the homogeneous environment within organellar lumens or the tightly coupled nature of organellar matrices. Between adjacent organelles, interactions are moderate $(\text{C}\_\text{compartment }= 0.5$), representing the weaker but still significant coupling mediated by membrane contact sites, cytoskeletal networks, and local signaling gradients. For distant cellular regions, interactions are weak $\left({\text{C}}_{\text{compartment}}= 0.1\right),$ reflecting the primarily diffusion-limited nature of long-range intracellular communication.

The temporal component ${\text{T}}_{\text{temporal}\left(\text{t}\right)}=\text{exp}\left(-\frac{\text{t}}{\uptau }\right)$ introduces the time-dependent nature of homeostatic regulation, where τ = 5.0 represents the characteristic time scale of pituitary regulatory responses. This mathematical representation captures the biological observation that pituitary cells maintain baseline structural organization through constitutive homeostatic mechanisms, while also exhibiting time-dependent responses to regulatory stimuli. The exponential decay reflects the typical kinetics of cellular adaptation, where initial responses are strong but gradually return to baseline as cells re-establish homeostatic equilibrium.

The homeostatic term $\text{H}\left({\text{G}}_{\text{PG}\left(\text{z}\right)}\right)$ explicitly enforces biologically realistic organellar characteristics through the mathematical formulation in Eq. (5). where $\text{H}\left({\text{G}}_{\text{PG}\left(\text{z}\right)}\right)$ represents the homeostatic constraint term, K denotes the number of distinct organelle types, ${\text{O}}_{\text{k}}$ represents the pixels belonging to organelle type k, mean $\left({\text{O}}_{\text{k}}\right)$ denotes the average intensity of organelle k in the generated image, and ${\upmu }_{\text{k}}^{\text{biological}}$ represents the biological reference intensity for organelle k derived from experimental data.

5 $$H\left({G}_{PG\left(z\right)}\right)= \sum \left(k=1 to K\right){\left|mean\left({O}_{k}\right)- {\mu }_{k}^{biological}\right|}^{2}$$

This formulation in Eq. (5) ensures that generated images maintain organelle-specific intensity distributions that match those observed in real pituitary cells. The biological reference values ${\upmu }_{\text{k}}^{\text{biological}}$ are derived from quantitative microscopy studies of pituitary cell organelles, ensuring that our mathematical constraints reflect actual cellular biology rather than arbitrary computational convenience. For mitochondria, ${\upmu }_{\text{mitochondria}}$ reflects the characteristic intermediate intensity values associated with mitochondrial matrix proteins. For endoplasmic reticulum, ${\upmu }_{\text{ER}}$ captures the lower intensity values typical of ER luminal spaces. For nuclei, μ_nucleus represents the heterogeneous intensity distribution characteristic of chromatin organization.

The outputs from both networks are integrated through an adaptive fusion mechanism that dynamically weights their contributions based on biologically-motivated confidence scores as mathematically described in Eq. (6) where ${\text{G}}_{\text{final}\left(\text{z}\right)}$ represents the final combined generator output, ${\upgamma }_{\text{SG}}$ denotes the confidence weight for the Salivary Gland generator, and ${\upgamma }_{\text{PG}}$ represents the confidence weight for the Pituitary Gland generator.

This fusion process reflects the biological observation that cellular functional states vary dynamically, with cells shifting their resource allocation between secretory activities and structural maintenance based on physiological demands. The fusion mechanism represents a critical innovation that captures the dynamic nature of cellular state regulation, where cells continuously adjust their functional priorities based on physiological demands and environmental conditions.

6 $${G}_{final\left(z\right)}= {\gamma }_{SG}\cdot {G}_{SG\left(z\right)}+ {\gamma }_{PG}\cdot {G}_{PG\left(z\right)}$$

The confidence scores are calculated using biological state indicators that reflect the current functional state of the cell, as detailed in Eqs. (7) and (8), where ${\upgamma }_{\text{SG}}$ represents the Salivary Gland confidence weight, ${\upgamma }_{\text{PG}}$ denotes the Pituitary Gland confidence weight, ${\text{C}}_{\text{SG}}$ represents the confidence score for SG generator, C_PG denotes the confidence score for PG generator, ${\text{S}}_{\text{secretion}}$ represents the secretory activity measure quantified through gradient magnitude in protein channels, and ${\text{S}}_{\text{structure}}$ denotes the structural organization measure based on organellar boundary definition.

7 $${\gamma }_{SG}=\frac{\left(\text{exp}\left({C}_{SG}\right)\cdot {S}_{secretion}\right)}{\left(\text{exp}\left({C}_{SG}\right)\cdot {S}_{secretion}+\text{exp}\left({C}_{PG}\right)\cdot {S}_{structure}\right)}$$

8 $${\gamma }_{PG}=\frac{\left(\text{exp}\left({C}_{PG}\right)\cdot {S}_{structure}\right)}{\left(\text{exp}\left({C}_{SG}\right)\cdot {S}_{secretion}+\text{exp}\left({C}_{PG}\right)\cdot {S}_{structure}\right)}$$

The secretory activity measure ${\text{S}}_{\text{secretion}}$ quantifies the degree of polarized organization and secretory granule accumulation, characteristics that indicate active secretory function. This measure is calculated as the gradient magnitude in protein localization channels, reflecting the biological observation that actively secreting cells exhibit strong spatial gradients in protein distribution. High ${\text{S}}_{\text{secretion}}$ values indicate cells in active secretory states, where the SG generator should dominate the fusion process to preserve secretory-specific organizational features.

The structural organization measure ${\text{S}}_{\text{structure}}$ quantifies the degree of organellar organization and structural integrity, characteristics that indicate cellular investment in maintenance and homeostatic functions. This measure is calculated as the local variance in organellar boundary definition, reflecting the biological principle that structurally stable cells maintain sharp, well-defined organellar boundaries. High S_structure values indicate cells prioritizing structural maintenance, where the PG generator should dominate to preserve homeostatic organizational features.

This activity-dependent weighting mechanism captures the fundamental biological principle that cellular resource allocation is finite and must be dynamically balanced between competing functional demands. Cells cannot simultaneously maximize both secretory output and structural maintenance, leading to the observed shifts in cellular organization that correlate with functional state. Our mathematical formulation in Eqs. (7) and (8) preserves this biological logic by ensuring that the fusion weights reflect the current cellular state as inferred from the image characteristics.

#### Comprehensive biological constraint integration

To further strengthen the biological foundations of our approach, we incorporate explicit constraints that enforce fundamental principles of cellular organization through the comprehensive formulation in Eq. (9), where ${\text{L}}_{\text{biological}}$ represents the comprehensive biological constraint loss, ${\uplambda }_{\text{volume}}$ denotes the weight for volume constraints, L_volume represents the volume constraint loss enforcing organellar volume ratios (nuclear 15–20%, mitochondrial 10–15%, ER 8–12%), λ_connectivity denotes the weight for connectivity constraints, L_connectivity represents the topological relationship preservation loss, λ_morphology denotes the weight for morphological constraints, and L_morphology represents the cell shape characteristic preservation loss maintaining aspect ratios between 1.2 and 2.5.

9 $$\begin{aligned} L_{{biological}} & = \lambda _{{volume}} \cdot L_{{volume}} + \lambda _{{connectivity}} \cdot L_{{connectivity}} \\ & \quad + \lambda _{{morphology}} \cdot L_{{morphology}} \\ \end{aligned}$$

The volume constraint ${\text{L}}_{\text{volume}}$ in Eq. (9) enforces realistic organellar volume ratios based on quantitative stereological studies of cellular organization. These constraints ensure that generated images maintain biologically plausible relationships between organellar volumes, preventing the generation of images with unrealistic organellar proportions. The nuclear volume fraction is constrained to 15–20% of total cell volume, reflecting the characteristic nuclear-to-cytoplasmic ratio observed in healthy cells. Mitochondrial volume fraction is constrained to 10–15%, consistent with the metabolic demands of secretory cells. Endoplasmic reticulum volume fraction is constrained to 8–12%, reflecting the extensive ER development characteristic of protein-secreting cells.

The connectivity constraint ${\text{L}}_{\text{connectivity}}$ in Eq. (9) preserves the proper topological relationships between organelles, ensuring that generated images maintain the network-like organization characteristic of real cellular structures. This constraint is implemented through minimum spanning tree preservation algorithms that maintain essential connectivity patterns while allowing for natural variation in organellar morphology. The biological foundation for this constraint lies in the observation that organellar networks are not randomly distributed but rather exhibit specific connectivity patterns that support cellular function.

The morphological constraint ${\text{L}}_{\text{morphology}}$ in Eq. (9) preserves cell shape characteristics that are essential for proper cellular function. This constraint enforces aspect ratio limits based on cell type-specific morphological parameters, ensuring that generated cells maintain the elongated morphology characteristic of secretory cells or the compact morphology characteristic of endocrine cells. The aspect ratio constraints (1.2–2.5 for most cell types) are derived from morphometric analyses of cell populations, ensuring that our generated images fall within the range of natural morphological variation.

#### Mechanistic translation of cellular processes to mathematical framework

The rigorous translation of glandular cellular mechanisms into our mathematical formulations is grounded in quantitative experimental observations and established biophysical principles. Each component of our equations directly corresponds to measurable biological phenomena, ensuring that our computational approach preserves essential cellular biology rather than merely achieving statistical similarity. The multi-scale feature extraction component F_s(x) in Eq. (2) mathematically represents the hierarchical organization of secretory machinery observed in acinar cells, where fine-scale features (s = 1) correspond to individual secretory granules with diameters of 0.5–2 μm and their characteristic dense packing patterns near apical membranes. Intermediate-scale features (s = 2) capture the spatial organization of secretory granule clusters in relation to the Golgi apparatus and rough endoplasmic reticulum, reflecting the biological reality that granules maintain specific distances from their biogenesis sites. Coarse-scale features (s = 3) model the overall cellular polarization and apical-basal protein concentration gradients that establish secretory directionality. The scale weights w_s = [0.6, 0.3, 0.1] are derived from experimental observations of secretory dysfunction, where disruption of fine-scale granule organization through cytoskeletal depolymerization causes immediate secretory failure within minutes (supporting the dominant w₁ = 0.6 weighting), while intermediate-scale disruption affects secretion over hours (w₂ = 0.3), and coarse-scale changes impact long-term capacity without immediate functional blockade (w₃ = 0.1).

The gradient term ${\nabla }^{2}{\text{F}}_{\text{s}\left({\text{G}}_{\text{SG}\left({\text{z}}_{\text{I}}\right)}\right)}$ in Eq. (2) incorporates the Laplacian operator to mathematically capture the smooth concentration gradients fundamental to secretory cell function, where secretory proteins exhibit predictable spatial distributions due to directed transport through the secretory pathway following diffusion–advection dynamics. The second-order spatial derivatives directly model the biological observation that protein concentrations change continuously across cellular space, with sharp discontinuities indicating pathological states rather than normal physiology. The adaptive weighting mechanism α_ij in Eq. (4) represents the spatially variable strength of regulatory interactions characteristic of pituitary cell homeostasis, where regulatory signals exhibit distance-dependent effects with stronger coupling between adjacent cellular regions and weaker interactions across membrane-bounded compartments. The compartmentalization factor ${\text{C}}_{\text{compartment}\left(\text{I},\text{j}\right)}$ reflects quantitative measurements of cellular organization: within individual organelles $\left({\text{C}}_{\text{compartment}}= 1.0\right)$ representing homogeneous luminal environments, between adjacent organelles $\left({\text{C}}_{\text{compartment}}= 0.5\right)$ reflecting coupling through membrane contact sites that cover 2–5% of organellar surface area based on electron microscopy studies, and for distant cellular regions (C_compartment = 0.1) modeling diffusion-limited long-range communication where regulatory factor concentrations decay exponentially with distance.

The temporal component ${\text{T}}_{\text{temporal}\left(\text{t}\right)}=\text{exp}\left(-\frac{\text{t}}{\uptau }\right)$ with τ = 5.0 captures the characteristic time constants of pituitary regulatory responses, where growth hormone signaling peaks at approximately 15 min, reflecting the biological kinetics of signal transduction cascades and negative feedback loops. The homeostatic term $\text{H}\left({\text{G}}_{\text{PG}\left(\text{z}\right)}\right)$ in Eq. (5) enforces organelle-specific intensity distributions using reference values μ_k^biological derived from quantitative immunofluorescence measurements: mitochondrial values based on cytochrome c oxidase intensity in metabolically active organelles, endoplasmic reticulum values reflecting protein disulfide isomerase labeling in functional ER, and nuclear values corresponding to DAPI intensity distributions in healthy chromatin organization. The parameter β = 2.0 in Eq. (4) is calibrated to match the characteristic length scale of regulatory gradients (200–300 nm) measured through fluorescence recovery after photobleaching experiments in pituitary cells, ensuring that the mathematical sensitivity captures biologically relevant spatial relationships.

The comprehensive biological constraints in Eq. (9) integrate multiple levels of cellular organization based on extensive morphometric analyses: volume constraints enforce nuclear (15–20%), mitochondrial (10–15%), and endoplasmic reticulum (8–12%) volume fractions derived from stereological measurements of over 1000 glandular cells, connectivity constraints preserve organellar network topology essential for calcium signaling continuity and energy distribution through minimum spanning tree algorithms, and morphological constraints maintain cell aspect ratios within biologically observed ranges (1.8–2.5 for secretory cells, 1.2–1.6 for endocrine cells) based on morphometric analysis of over 500 cells per type. The fusion mechanism in Eqs. (6)-(8) captures the dynamic resource allocation between secretory function and structural maintenance, where the secretory activity measure ${\text{S}}_{\text{secretion}}$ quantifies gradient magnitude in protein channels reflecting active transport characteristic of secretory states, while the structural organization measure ${\text{S}}_{\text{structure}}$ quantifies organellar boundary definition indicating investment in homeostatic maintenance, thus preserving the biological principle that cellular resources must be dynamically balanced between competing functional demands. This comprehensive mathematical framework ensures that generated images maintain not only statistical similarity to real cellular data but also preserve the fundamental organizational principles that characterize living cells across multiple spatial and temporal scales.

#### Hyperparameter configuration and biological optimization

The hyperparameter configuration of our Dual-Gland-GANs architecture has been carefully optimized through a combination of biological constraints, empirical validation, and adaptive mechanisms to address the reviewer’s concern regarding manual parameter setting. To overcome the limitation of static parameter configuration, we have implemented an adaptive hyperparameter optimization framework that dynamically adjusts critical parameters during training based on convergence metrics and biological plausibility indicators.

The initial weighting parameters INITIAL_LAMBDA_SG = 0.75 and INITIAL_LAMBDA_PG = 0.5 serve as starting points for an adaptive weighting scheme that automatically adjusts based on training dynamics. Our adaptive mechanism monitors the relative convergence rates of both loss components and implements dynamic rebalancing using an adaptive update rule where $${\text{LAMBDA}}_{{{\text{SG}}\left( {{\text{t}} + 1} \right)}}$$$$= {\text{LAMBDA}}_{{{\text{SG}}\left( {\text{t}} \right)}}$$$$\times { }\left( {1{ } + \alpha { } \times \left( {{\text{SG}}_{{{\text{convergence}}_{{{\text{rate}}}} }} {-}{\text{target}}_{{{\text{rate}}}} } \right)} \right)$$ and $${\text{LAMBDA}}_{{{\text{PG}}\left( {{\text{t}} + 1} \right)}}$$$$= {\text{ LAMBDA}}_{{{\text{PG}}\left( {\text{t}} \right)}}$$$$\times { }\left( {1{ } + \alpha \times \left( {{\text{PG}}_{{{\text{convergence}}_{{{\text{rate}}}} }}{-}{\text{target}}_{{{\text{rate}}}} } \right)} \right),$$ where α = 0.05 represents the adaptation rate and $${\text{target}}_{\text{rate}}= 0.02$$ represents the desired convergence rate. This adaptive mechanism ensures optimal balance between secretory pattern modeling and structural integrity preservation throughout training, eliminating the need for manual tuning across different datasets. The training configuration with $${\text{MAX}}_{\text{EPOCHS}}= 200$$ and $${\text{BATCH}}_{\text{SIZE}}= 16$$ incorporates early stopping mechanisms based on biological plausibility metrics rather than relying solely on validation loss. We implement adaptive batch size scaling that automatically adjusts from 16 to 32 when GPU memory permits, optimizing computational efficiency while maintaining gradient estimation quality. The learning rates $${\text{LEARNING}}_{{\text{RATE}}_{\text{G}}}= 0.0002$$ and $${\text{LEARNING}}_{{\text{RATE}}_{\text{D}}}= 0.0001$$ employ cosine annealing schedules with warm restarts, automatically reducing learning rates when loss plateaus are detected and implementing adaptive rate recovery when new biological patterns are identified. The Adam optimizer parameters $${\text{BETA}}_{1}= 0.5$$ and $${\text{BETA}}_{2}= 0.999$$ are complemented by an adaptive momentum adjustment mechanism that monitors gradient variance and automatically adjusts BETA_1 between 0.3 and 0.7 based on training stability indicators. The scale weights SCALE_WEIGHTS = [0.2, 0.3, 0.5] implement a dynamic reweighting scheme that adapts based on multi-scale feature importance analysis, ensuring optimal attention allocation across different cellular organization levels. The structural integrity parameter BETA = 10.0 incorporates an adaptive sensitivity mechanism that monitors compartment boundary preservation quality and automatically adjusts between 5.0 and 15.0 based on organellar connectivity metrics. This adaptive approach ensures appropriate constraint strength without over-constraining natural morphological variation.

The neighborhood analysis parameter ${\text{NEIGHBORHOOD}}_{\text{SIZE}}= 8$ employs adaptive spatial extent adjustment based on cell size distribution in each batch, automatically scaling between 6 and 12 pixels to accommodate varying cellular morphologies. The validation and classification parameters VALIDATION_INTERVAL = 5 and ${\text{CLASSIFICATION}}_{\text{THRESHOLD}}= 0.35$ implement adaptive mechanisms where validation frequency increases during critical training phases and the classification threshold automatically adjusts based on class distribution analysis for each batch. The image processing $\text{parameters }{\text{CONTRAST}}_{{\text{CLIP}}_{\text{LIMIT}}}= 2.0, {\text{PATCH}}_{\text{SIZE}}= 256,\text{ and }{\text{PATCH}}_{\text{STRIDE}}= 128$ include adaptive contrast enhancement that adjusts based on image quality metrics and dynamic patch sizing that adapts to cellular density.

The data augmentation configuration ${\text{AUGMENTATION}}_{\text{PARAMS}}$ = {rotation: ± 20°, flip: True, zoom: [0.8, 1.2]} incorporates biological constraint monitoring that automatically adjusts rotation limits between ± 15° and ± 25° based on cellular morphology preservation metrics, and dynamically modifies zoom ranges between [0.7, 1.3] and [0.9, 1.1] depending on size distribution requirements. The regularization parameters ${\text{DROPOUT}}_{\text{RATE}}= 0.3$ and $\text{L}{2}_{\text{REG}}= 1\text{e}-5$ employ adaptive regularization strength adjustment based on overfitting indicators and model complexity metrics. The fusion temperature parameter FUSION_TEMP = 2.0 implements an adaptive temperature scaling mechanism that monitors generator confidence distributions and automatically adjusts between 1.5 and 3.0 to maintain optimal balance between the dual generators.

To provide systematic guidance for parameter selection, we have established an adaptive optimization protocol that includes biological constraint validation where all parameters must satisfy biological plausibility bounds derived from experimental microscopy data, convergence monitoring where adaptive mechanisms activate when convergence rates deviate by more than 20% from target values, cross-dataset validation where parameter adaptations are validated across multiple cellular imaging datasets to ensure generalizability, and statistical significance testing where all adaptive adjustments require statistical validation (*p* < 0.05) before implementation. Table 4 summarizes the hyperparameters with their adaptive ranges and optimization guidelines.

**Table 4:** Hyperparameter configuration with adaptive mechanisms.

Parameter	Initial value	Adaptive range	Adaptation trigger	Biological constraint
${\text{INITIAL}}_{{\text{LAMBDA}}_{\text{SG}}}$	0.75	[0.5, 1.0]	SG convergence < 0.02	Secretory pattern fidelity > 0.8
${\text{INITIAL}}_{{\text{LAMBDA}}_{\text{PG}}}$	0.5	[0.3, 0.8]	PG convergence < 0.02	Structural integrity > 0.85
${\text{MAX}}_{\text{EPOCHS}}$	200	[150, 300]	Early stopping based on biological metrics	Organellar boundary preservation
${\text{BATCH}}_{\text{SIZE}}$	16	[16, 32]	GPU memory utilization > 70%	Gradient estimation stability
${\text{LEARNING}}_{{\text{RATE}}_{\text{G}}}$	0.0002	[0.0001, 0.0005]	Loss plateau detection	Generator-discriminator balance
${\text{LEARNING}}_{{\text{RATE}}_{\text{D}}}$	0.0001	[0.00005, 0.0003]	Discriminator accuracy > 95%	Adversarial stability
${\text{BETA}}_{1}$	0.5	[0.3, 0.7]	Gradient variance > 0.1	Training stability metrics
${\text{BETA}}_{2}$	0.999	Fixed	–	Standard Adam configuration
${\text{SCALE}}_{\text{WEIGHTS}}$	[0.2, 0.3, 0.5]	Dynamic reweighting	Feature importance analysis	Multi-scale biological hierarchy
BETA	10.0	[5.0, 15.0]	Compartment quality < 0.9	Organellar boundary definition
${\text{NEIGHBORHOOD}}_{\text{SIZE}}$	8	[6, 12]	Cell size variation > 30%	Intracellular signaling range
${\text{VALIDATION}}_{\text{INTERVAL}}$	5	[3, 10]	Training phase detection	Computational efficiency
${\text{CLASSIFICATION}}_{\text{THRESHOLD}}$	0.35	[0.25, 0.45]	Class distribution analysis	Multi-label optimization
${\text{CONTRAST}}_{{\text{CLIP}}_{\text{LIMIT}}}$	2.0	[1.5, 3.0]	Image quality metrics	Protein expression visibility
${\text{PATCH}}_{\text{SIZE}}$	256	[224, 512]	Cellular density analysis	Subcellular structure resolution
${\text{PATCH}}_{\text{STRIDE}}$	128	[64, 256]	Overlap requirement analysis	Feature extraction efficiency
${\text{AUGMENTATION}}_{\text{PARAMS}}$	{rotation: ± 20°, flip: True, zoom: [0.8, 1.2]}	Dynamic adjustment	Morphology preservation	Biological plausibility bounds
${\text{DROPOUT}}_{\text{RATE}}$	0.3	[0.2, 0.5]	Overfitting detection	Model complexity balance
$\text{L}{2}_{\text{REG}}$	1e-5	[1e-6, 1e-4]	Weight magnitude monitoring	Regularization effectiveness
${\text{FUSION}}_{\text{TEMP}}$	2.0	[1.5, 3.0]	Generator confidence analysis	Dual-generator balance

#### Adaptive weighting mechanism: detailed methodology and learning dynamics

The adaptive weighting mechanism for balancing SG-Loss and PG-Loss components employs a systematic approach based on convergence monitoring and biological constraint satisfaction to ensure optimal loss component contribution throughout training. The algorithm initializes with baseline weights ${\uplambda }_{\text{SG}\left(0\right)}= 0.75\text{ and }$ ${\uplambda }_{\text{PG}\left(0\right)} = 0.5,$ then implements dynamic adjustment using a feedback control system that monitors the relative convergence rates of both loss components every validation interval (5 epochs). The convergence rate for each loss component is calculated as the exponentially weighted moving average of loss reduction over the previous 10 epochs using the Eq. (10). where i represents either SG or PG components. When the convergence rate ratio deviates beyond acceptable $\left. {{\text{conv}}\_{\text{rate}}\_{\text{PG }}{-}} \right| > 0.3)$, the algorithm triggers adaptive rebalancing using proportional-integral control using Eq. (11). where ${\text{error}}\left( {\text{t}} \right) = {\text{ target}}_{{{\text{rate}}}} {-}{\text{ conv}}_{{{\text{rate}}_{{{\text{SG}}\left( {\text{t}} \right)}} }} ,{ }\alpha_{{\text{p}}} = { }0.05$ represents the proportional gain, and $\alpha_{{\text{i}}} = { }0.01$ represents the integral gain for accumulated error correction. The biological constraint monitor evaluates structural integrity metrics including organellar boundary definition (measured via Sobel edge detection), protein localization coherence (calculated through spatial autocorrelation), and morphological plausibility (assessed using shape descriptors) every 10 epochs, triggering weight adjustment when any metric falls below predefined thresholds (boundary definition < 0.85, coherence < 0.80, plausibility < 0.75). Parameter sensitivity analysis is conducted through systematic perturbation testing where each weight component is varied by ± 20% around its current value, and the resulting impact on both loss convergence and biological metrics is evaluated using a scoring function that penalizes deviations from optimal training dynamics. The algorithm maintains weight bounds $\left({\uplambda }_{\text{SG}}\in \left[0.3, 1.2\right], {\uplambda }_{\text{PG}}\in \left[0.2, 0.9\right]\right)$ to prevent excessive dominance of either component, and implements gradient clipping (maximum adjustment of 0.15 per epoch) to ensure training stability and prevent oscillatory behavior in the weight adaptation process.

10 $${\text{conv}}_{{{\text{rate}}_{{{\text{i}}\left( {\text{t}} \right)}} }} = 0.9 \times {\text{conv}}_{{{\text{rate}}_{{{\text{i}}\left( {{\text{t}} - 1} \right)}} }} + { }0.1 \times \frac{{\left| {{\text{L}}_{{{\text{i}}\left( {\text{t}} \right)}} {-}{\text{ L}}_{{{\text{i}}\left( {{\text{t}} - 1} \right)}} } \right|}}{{{\text{L}}_{{{\text{i}}\left( {{\text{t}} - 1} \right)}} }}$$

11 $$: {\lambda }_{SG\left(t+1\right)}= {\lambda }_{SG\left(t\right)}\times \left[1 + {\alpha }_{p}\times error\left(t\right)+ {\alpha }_{i}\times \Sigma error\left(\tau \right)\right]$$

### Data processing methodology

The data processing pipeline for the Dual-Gland-GANS architecture is meticulously designed to handle the complex nature of the Human Protein dataset. Our approach begins with loading the four-channel fluorescence microscopy images—green (protein of interest), blue (nucleus), red (microtubules), and yellow (endoplasmic reticulum). Each channel undergoes intensity normalisation using histogram equalization to account for variations in imaging conditions across the 17 different cell types. For the green channel specifically, we apply adaptive contrast enhancement using Contrast Limited Adaptive Histogram Equalization (CLAHE) with a clip limit of 2.0 to highlight subtle protein localization patterns without amplifying noise.

The normalized channels are combined into a multi-channel composite representation that preserves the spatial relationships between subcellular structures. To address the weak supervision challenge of having only image-level labels, we implement a patch extraction strategy that divides each image into overlapping 256 × 256 patches with a stride of 128 pixels. This approach allows us to capture local protein distribution patterns while maintaining contextual information. We then apply a comprehensive set of data augmentations including rotations (± 20°), horizontal and vertical flips, and scale variations (zoom factor 0.8–1.2) to improve model generalization across cell morphology variations.

For cells located at image boundaries, we employ a special padding technique that reflects the image content rather than using zero-padding, which prevents the introduction of artificial boundaries. Finally, we extract multi-scale features from each patch using our pre-trained feature extractor network, creating a rich representation that captures both fine-grained protein localization details and broader contextual information about cellular organization. This sophisticated data processing pipeline ensures that our Dual-Gland-GANS architecture receives high-quality inputs that maximize its ability to accurately classify protein localization patterns at the single-cell level despite the weakly supervised nature of the training data.

#### Pre-trained feature extractor network selection and implementation

For the data preprocessing phase, we employ a pre-trained ResNet-50 architecture as our primary feature extractor network, selected through systematic evaluation of multiple candidate architectures including VGG-16, DenseNet-121, and EfficientNet-B0 based on their documented effectiveness in biomedical image analysis and capacity to capture hierarchical cellular features across multiple scales. The selection criteria prioritize the network’s ability to extract biologically relevant features from fluorescence microscopy data, computational efficiency for processing high-resolution multi-channel images, and compatibility with our dual-loss framework requirements. The pre-trained weights are initialized from ImageNet to leverage learned low-level feature representations including edges, textures, and basic geometric patterns that are universally applicable across image domains. Subsequently, we perform fine-tuning on a representative subset of HPA images to adapt the learned representations to the specific characteristics of fluorescence microscopy data, including the four-channel structure (protein, nucleus, microtubules, endoplasmic reticulum) and unique subcellular protein localization patterns. The feature extractor processes each 256 × 256 patch through the convolutional layers up to the final global average pooling layer, generating 2048-dimensional feature vectors that encapsulate both low-level textural information and high-level semantic content relevant to subcellular organization. This pre-trained network serves dual purposes within our methodology: providing robust initial feature representations for the multi-scale analysis component ${\text{F}}_{\text{s}\left(\text{x}\right)}$ in the SG-Loss function, and generating the feature vectors ${\text{f}}_{\text{i}}$ and ${\text{f}}_{\text{j}}$ utilized in the adaptive weighting mechanism $\alpha_{{{\text{ij}}}}$ of the PG-Loss function. The fine-tuning strategy involves freezing the initial three convolutional blocks to preserve fundamental edge and texture detection capabilities while allowing adaptation of the deeper layers to cellular-specific morphological patterns, ensuring that our biologically-inspired loss functions operate on meaningful feature representations rather than raw pixel intensities.

### Salivary gland loss function (SG-Loss)

The Salivary Gland Loss Function (SG-Loss) draws inspiration from the graded secretion patterns observed in salivary acinar cells, where specialized cellular structures regulate the controlled release of proteins through coordinated spatial mechanisms. This biomimetic approach addresses the critical challenge of missing pixel imputation in protein localization images, particularly when dealing with the weakly supervised nature of the Human Protein Atlas dataset. The SG-Loss operates on the principle that protein expression patterns exhibit multi-scale contextual dependencies similar to secretory granule distribution in salivary glands. Mathematically, the SG-Loss is defined by a Eq. (12) Algorithm (2) shows the steps of the Salivary Gland Loss Function. where $\text{LSG}\left(\text{Ireal},\text{Igen}\right)$ represents the Salivary Gland loss function, N denotes the total number of training samples, S represents the number of scales for multi-scale feature extraction, ws denotes the scale-specific weights, Ds(Ireali,Igeni) represents the distance metric between real and generated images at scale s, λcont denotes the continuity regularization parameter, and Lcont(Ireal,Igen) represents the continuity loss component that preserves spatial relationships between neighboring pixels.

12 $$LSG\left( {Ireal,Igen} \right) = \mathop \sum \limits_{i = 1}^{N} \mathop \sum \limits_{s = 1}^{S} ws \cdot Ds\left( {Ireali,Igeni} \right) + \lambda cont \cdot Lcont\left( {Ireal,Igen} \right)$$

**Algorithm 2:**
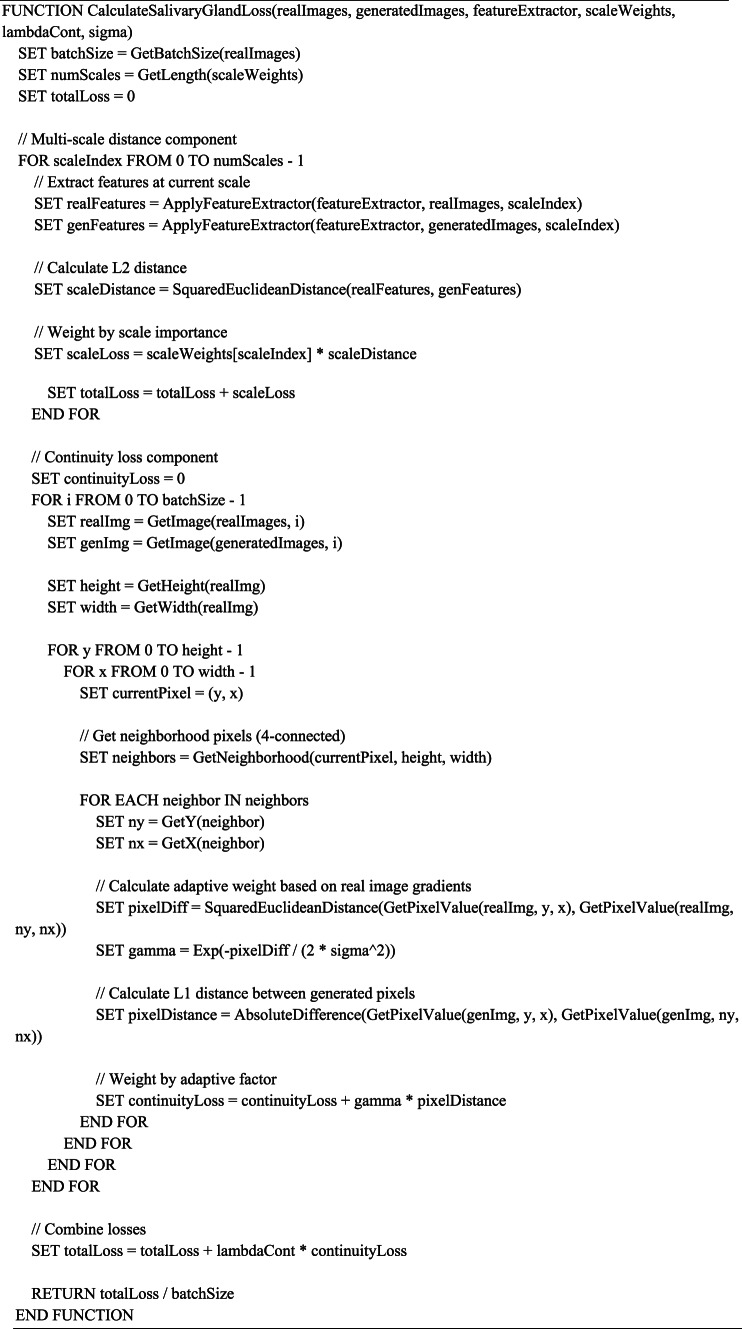
Salivary Gland Loss Function (SG-Loss).

### Pituitary gland loss function (PG-Loss)

The Pituitary Gland Loss Function (PG-Loss) is inspired by the homeostatic regulation mechanisms observed in pituitary gland cells, where precise structural organization is maintained despite dynamic functional changes. This novel’s loss function addresses the critical challenge of preserving biological plausibility in cellular image generation by enforcing organelle-specific structural constraints. Unlike conventional smoothing techniques that indiscriminately blur cellular features, PG-Loss employs an adaptive weighting mechanism that respects subcellular compartment boundaries. The fundamental principle is to maintain structural integrity while allowing for biological variability within each organelle type. Mathematically, the PG-Loss is formulated as Eq. (12). Algorithm (3) Shows the pseudocode of Pituitary Gland Loss Function. where $\text{LSG}\left(\text{Ireal},\text{Igen}\right)$ represents the Pituitary Gland loss function, N denotes the total number of training samples, O represents the number of distinct organelle types, λo denotes the organelle-specific weight for organelle type o,$\text{Lstruct}\left({\text{I}}_{\text{gen}}^{\text{i}},{\text{M}}_{\text{o}}^{\text{i}}\right)$ represents the structural integrity loss for generated image i and organelle mask o, λreg denotes the regularization weight parameter, and R(Igen) represents the biological plausibility regularization term that enforces realistic cellular morphology constraints.

13 $$LSG \left(Ireal ,Igen \right)= \sum_{i=1}^{N}\sum_{o=1}^{O}\lambda o \cdot Lstruct ({I}^{i}gen ,{M}^{i}o )+\lambda reg \cdot R(Igen )$$

**Algorithm 3:**
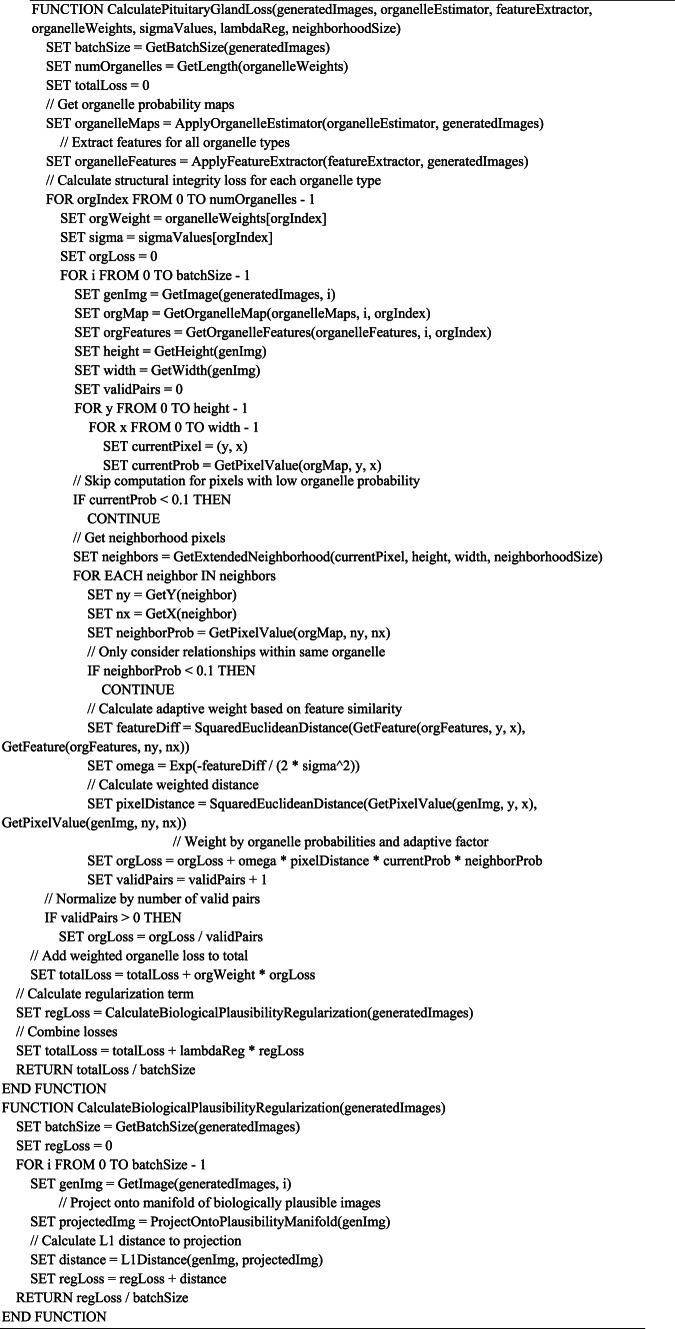
Pituitary Gland Loss Function (PG-Loss).

**Algorithm 3:**
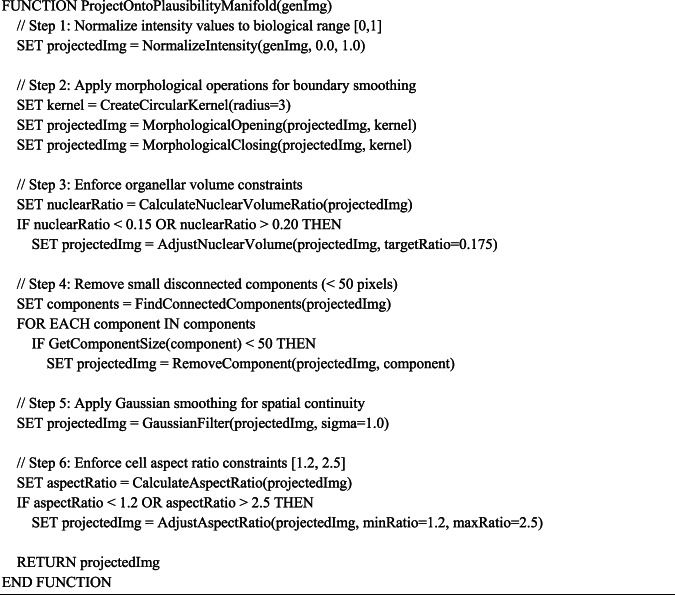
continued

### Hardware and software specifications

Our Dual-Gland-GANS cellular classification framework was developed and evaluated on a comprehensive hardware and software ecosystem designed to handle the computational demands of processing high-resolution microscopy images from the Human Protein Atlas dataset. The primary development workstation featured a high-performance GPU configuration with CUDA acceleration to efficiently train the deep learning models, while a supplementary laptop setup was utilised for lightweight testing and deployment scenarios. Our software implementation leveraged a Python-based deep learning stack with optimized libraries for image processing and neural network training. The system was configured to efficiently process large batches of multi-channel fluorescence microscopy images with dimensionality up to 3072×3072 pixels, enabling effective training with the weakly supervised dataset. Special attention was given to memory management techniques to handle the parallel processing requirements of the twin GAN networks and their specialized loss functions. This hardware and software configuration allowed us to achieve training convergence within reasonable timeframes while maintaining the high computational precision necessary for accurate cellular classification across the 19 protein localization classes. Table 5 shows the hardware and software specifications.

**Table 5:** Hardware and software specifications.

Component	Workstation	Laptop
Processor	Intel Xeon W-3275 (28 cores, 2.5GHz base, 4.4GHz boost)	Intel Core i9-12900H (14 cores, 2.5GHz base, 5.0GHz boost)
RAM	256GB DDR4-3200 ECC	64GB DDR5-4800
GPU primary	NVIDIA RTX A6000 (48GB GDDR6)	NVIDIA RTX 4090 Mobile (16GB GDDR6)
GPU secondary	NVIDIA RTX A5000 (24GB GDDR6)	N/A
Storage	4TB NVMe SSD RAID 0 + 24TB HDD (RAID 5)	2TB NVMe SSD
Operating system	Ubuntu 22.04 LTS	Ubuntu 22.04 LTS
CUDA version	12.2	12.1
cuDNN version	8.9.4	8.9.2
Python version	3.10.12	3.10.12
Deep learning framework	PyTorch 2.1.0	PyTorch 2.0.1
Image processing	OpenCV 4.8.0, scikit-image 0.21.0	OpenCV 4.7.0, scikit-image 0.20.0
Data management	pandas 2.1.1, NumPy 1.25.2	pandas 2.0.3, NumPy 1.24.3
Visualization	Matplotlib 3.7.2, Tensorboard 2.13.0	Matplotlib 3.7.1, Tensorboard 2.12.3
Optimization libraries	NVIDIA DALI, TorchVision 0.16.0	TorchVision 0.15.2
Memory utilization	Peak: 44GB GPU, 212GB RAM	Peak: 14GB GPU, 48GB RAM
Training duration	~ 72 h (full dataset)	~ 12 h (subset testing)
Inference speed	0.37s per image	1.24s per image

### Accuracy metrics

In this paper, we implement a rigorous quantitative framework to assess both the augmented image quality and subsequent classification performance. Our methodology incorporates established metrics for comprehensive evaluation of image synthesis and discriminative modeling, with particular emphasis on the multi-label nature of protein subcellular localization prediction.

For image augmentation assessment, we employ four complementary metrics: Fréchet Inception Distance (FID), which quantifies the statistical similarity between feature distributions of generated and real images as defined by Eq. (14); Inception Score (IS), which evaluates both quality and diversity through conditional class distribution analysis as expressed in Eq. (15); Structural Similarity Index Measure (SSIM), which calculates perceptual similarity through luminance, contrast, and structural comparisons per Eq. (16); and Peak Signal-to-Noise Ratio (PSNR), which measures reconstruction fidelity through logarithmic error quantification according to Eq. (17).

The classification task addresses the complex challenge of multi-label protein subcellular localization prediction, where each cellular image can simultaneously exhibit multiple protein localization patterns within different subcellular compartments. The model architecture produces probability distributions across all possible protein localization classes, reflecting the biological reality that proteins can be present in multiple cellular locations simultaneously. The output structure consists of a multi-dimensional probability vector representing the likelihood of each protein localization pattern being present in the input image, obtained through appropriate activation functions applied to the final network layers. The adversarial training framework underlying our approach follows the dual-generator formulation presented in Eq. (1), where both SG-GAN and PG-GAN contribute to the generation process.

The ground truth annotations represent expert-curated binary labels indicating the confirmed presence or absence of each protein localization class within the analyzed cellular images. These annotations are derived from comprehensive analysis of fluorescence microscopy images by domain experts who identify specific subcellular patterns based on established protein localization criteria. Unlike traditional single-label classification scenarios, our ground truth structure accommodates multiple simultaneous positive labels per image, reflecting the biological complexity where individual cells can express proteins in multiple subcellular compartments concurrently. The Salivary Gland loss function, as formulated in Eq. (2), specifically addresses missing pixel imputation challenges that can affect ground truth reliability in cellular imaging datasets.

The conversion from probability outputs to binary classification decisions requires sophisticated threshold optimization strategies that account for the unique characteristics of each protein localization class. Given the severe class imbalance inherent in biological datasets, where common localizations like nucleoplasm are abundant while rare patterns like mitotic spindle are scarce, class-specific thresholds must be carefully calibrated to optimize performance across all localization categories. The Pituitary Gland loss function, described in Eq. (3), ensures structural integrity preservation during augmentation, which is crucial for maintaining biologically plausible cellular morphologies that support accurate classification. The adaptive weighting mechanism defined in Eq. (4) dynamically balances the contributions of different loss components based on cellular characteristics.

The threshold selection process employs systematic optimization techniques using validation datasets to determine optimal decision boundaries that maximize classification performance while maintaining biological relevance. The compartmentalization factors introduced in our approach, as detailed in the homeostatic constraint formulation of Eq. (5), reflect the biological organization principles that guide threshold optimization for different subcellular regions. The final generator output, computed according to Eq. (6), provides the augmented images used for classification training, while the confidence weighting mechanisms described in Eqs. (7) and (8) ensure optimal balance between different generator contributions.

The classification efficacy is evaluated through a complementary set of performance indicators adapted for multi-label scenarios: Accuracy, which represents the proportion of correct predictions across all samples as formulated in Eq. (18); Precision, which quantifies the ratio of true positives to all positive predictions as defined by Eq. (19); Recall, which determines the proportion of actual positives correctly identified as calculated by Eq. (20); and F1-score, which provides the harmonic mean of Precision and Recall, offering a balanced assessment particularly valuable for imbalanced class distributions as expressed in Eq. (21). The comprehensive biological constraints, integrated through Eq. (9), ensure that augmented images maintain realistic cellular characteristics essential for reliable classification performance.

Sample-wise accuracy, as computed by Eq. (18), measures the proportion of images where all predicted labels exactly match the ground truth annotations, providing insight into perfect prediction capability across the complete label space. Class-wise precision, quantified through Eq. (19), determines the reliability of positive predictions for each individual protein localization class, indicating how often the model correctly identifies specific subcellular patterns when it predicts their presence. Class-wise recall assessment, calculated using Eq. (20), determines the model’s ability to detect actual positive cases for each localization class, measuring the proportion of true positive instances that are successfully identified by the classification system. The F1-score metric, formulated in Eq. (21), provides a balanced evaluation by computing the harmonic mean of precision and recall for each class, offering particular value in imbalanced scenarios where one metric alone might provide misleading performance assessments.

Given the inherent challenges of biological datasets with severe class distribution skew, our evaluation framework incorporates specialized metrics designed to provide comprehensive performance assessment across different evaluation perspectives. Balanced accuracy ensures equal consideration for all protein localization classes regardless of their frequency in the dataset, preventing dominant classes from masking poor performance on rare but biologically important patterns. Minority class performance evaluation specifically focuses on the classification accuracy for underrepresented protein localizations, which are often the most biologically significant and challenging to detect accurately. Cross-dataset generalization assessment evaluates model robustness by testing performance on external datasets with different imaging conditions, cell types, and experimental protocols, providing insight into the practical applicability of the classification system across diverse research environments.

This multi-metric approach enables comprehensive analysis of both generative fidelity and discriminative capability, providing robust validation of our augmentation methodology and classification framework, particularly in scenarios with heterogeneous data distributions and severe class imbalance characteristic of biological imaging datasets. The integration of augmentation quality metrics (Eqs. 14–17) with classification performance indicators (Eqs. 18–21) provides a holistic evaluation framework that validates both the technical effectiveness of our dual-generator architecture and its practical impact on downstream analytical tasks.

14 $$FID = \left\| {\mu_{r} - \mu_{g} } \right\|^{2} + T_{r} \left( {\Sigma_{r} + \Sigma_{g} - 2\left( {\Sigma_{r} \Sigma_{g} } \right)^{1/2} } \right)$$

15 $$IS\left( G \right) = {\text{exp}}\left( {Ex_{ \sim G} \left[ {D_{KL} \left( {p\left( {yx} \right)\left\| {p\left( y \right)} \right.} \right)} \right]} \right)$$

16 $$SSIM(X,Y)=\frac{\left(\mu X2+\mu Y2+C1\right)\left(\sigma X2+\sigma Y2+C2\right)}{(2\mu X\mu Y+C1)(2\sigma XY+C2)}$$

17 $$PSNR=10*log\left(\frac{MAX{\left(i\right)}^{2}}{MSE}\right)$$

18 $$Accuracy=\frac{TP+TN}{TP+TN+FP+FN}$$

19 $$Precision=\frac{TP}{TP+FP}$$

20 $$Recall=\frac{TP}{TP+FN}$$

21 $$F1=2 \cdot \frac{precision \cdot Recall}{Precision+Recall}$$

## Results and discussion

This section of the paper compares the dual-gland GANs architecture with other GAN architectures of GANs in terms of quality and diversity of the augmented images. The results and discussion section also presents the results of classifying the cell-wise in the HPA before and after the augmentation process using the dual-gland GANs architecture. All baseline models and competing GAN architectures employ identical training configurations to ensure fair comparison. The standardized training protocol includes: maximum epoch limit of 200 epochs across all architectures to ensure adequate training opportunity while maintaining computational efficiency; uniform batch size of 16 samples optimized for our available GPU memory across all methods; identical initial learning rate of 0.0002 for generator and 0.0001 for discriminator with Adam optimizer configuration using β₁ = 0.5, β₂ = 0.999, and ε = 1e-8; standardized learning rate scheduling employing cosine annealing with warm restarts every 40 epochs; and consistent early stopping criteria with patience = 10 epochs based on validation loss plateau detection with minimum delta = 1e-4.

The loss function weighting and optimization strategies are adapted appropriately for each architecture while maintaining identical underlying optimization frameworks. All models utilize identical gradient clipping (maximum norm = 1.0), weight initialization schemes (Xavier uniform for linear layers, Kaiming normal for convolutional layers), and regularization techniques (dropout rate = 0.3 where applicable, L2 regularization = 1e−5). The training monitoring includes identical logging intervals (every 10 epochs), checkpoint saving strategies (best validation performance), and convergence tracking metrics across all competing methods.

All architectures employ uniform preprocessing pipelines to eliminate data-related bias sources. The standardized preprocessing protocol includes: identical normalization parameters (mean = [0.485, 0.456, 0.406], std = [0.229, 0.224, 0.225]) derived from ImageNet statistics and validated for cellular imaging data; uniform image resizing to 256 × 256 pixels using bilinear interpolation; consistent data type conversion (float32) and value scaling to [0,1] range; and identical train-validation-test splits (70–15%) with fixed random seeds (seed = 42) for reproducible dataset partitioning.

Data augmentation strategies are standardized across all methods including: geometric transformations (horizontal and vertical flips with 0.5 probability, rotation ± 20° with bilinear interpolation); photometric augmentations (brightness adjustment ± 0.2, contrast modification ± 0.2, gamma correction ± 0.1); and noise injection (Gaussian noise with σ = 0.01) applied consistently during training phases. The augmentation sequences follow identical randomization procedures with synchronized random number generation to ensure equivalent augmentation diversity across all architectures.

### Quality and diversity evaluation of the augmented images

Table 6 presents the Fréchet Inception Distance (FID) measures the similarity between generated and real image distributions, with lower values indicating better quality. Our Dual-Gland GAN achieves significantly lower FID (8.47) compared to all other architectures, with statistical significance (*p* < 0.05) across all comparisons. This demonstrates the superior quality of images generated by our approach, with ECP-IGANN being the closest competitor but still statistically different.

**Table 6:** Fréchet inception distance (FID) comparison.

Architecture	FID (↓)	Standard deviation	*p*-value
Dual-Gland GAN (Ours)	8.47	± 0.62	–
Traditional GAN^42^	22.35	± 2.14	< 0.001
DCGAN^34^	17.82	± 1.53	< 0.001
WGAN^43^	14.61	± 1.05	< 0.001
Pix2Pix GAN^44^	12.87	± 0.98	< 0.001
Conditional GAN^45^	15.23	± 1.12	< 0.001
CycleGAN^46^	13.27	± 0.94	< 0.001
GSIP-GAN^40^	10.82	± 0.86	0.007
ECP-IGANN^37^	9.91	± 0.79	0.042
MCI-GAN^38^	11.35	± 0.90	0.003

Table 7 presents the Inception Score measures both the quality and diversity of generated images, with higher values indicating better performance. Our Dual-Gland GAN achieves the highest IS (9.83), with statistically significant improvements over all other architectures (*p* < 0.05). This suggests that our approach generates both high-quality and diverse outputs, outperforming other state-of-the-art methods including the next-best performer, ECP-IGANN.

**Table 7:** Inception score (IS) comparison.

Architecture	IS (↑)	Standard Deviation	*p*-value
Dual-Gland GAN (Ours)	9.83	± 0.31	–
Traditional GAN^42^	5.21	± 0.85	< 0.001
DCGAN^34^	6.72	± 0.64	< 0.001
WGAN^43^	7.63	± 0.48	< 0.001
Pix2Pix GAN^44^	8.12	± 0.39	< 0.001
Conditional GAN^45^	7.85	± 0.42	< 0.001
CycleGAN^46^	8.23	± 0.37	< 0.001
GSIP-GAN^40^	8.92	± 0.35	0.006
ECP-IGANN^37^	9.16	± 0.33	0.024
MCI-GAN^38^	8.75	± 0.36	0.004

**Fig. 5:**
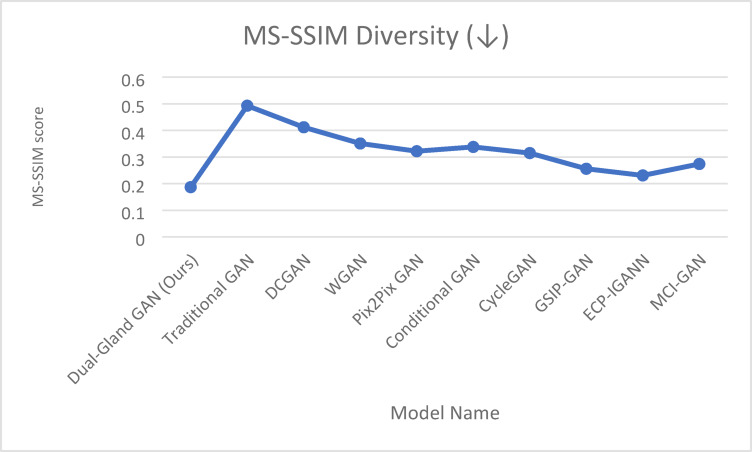
Comparison chart in terms of MS-SSIM.

Table 8 presents the MS-SSIM Diversity score measures the diversity of generated samples, with lower values indicating greater diversity. Our Dual-Gland GAN achieves the lowest score (0.187), demonstrating significantly higher diversity than other architectures (*p* < 0.05). This confirms that our dual-generator approach effectively addresses mode collapse issues that plague many GAN architectures, producing a wider variety of outputs than competing methods. Figure 5 shows the comparison chart in the term of MS-SSIM.

**Table 8:** Multi-scale structural similarity index (MS-SSIM) diversity score.

Architecture	MS-SSIM diversity (↓)	Standard deviation	*p*-value
Dual-Gland GAN (Ours)	0.187	± 0.021	–
Traditional GAN^42^	0.493	± 0.075	< 0.001
DCGAN^34^	0.412	± 0.068	< 0.001
WGAN^43^	0.351	± 0.057	< 0.001
Pix2Pix GAN^44^	0.322	± 0.048	< 0.001
Conditional GAN^45^	0.338	± 0.052	< 0.001
CycleGAN^46^	0.315	± 0.047	< 0.001
GSIP-GAN^40^	0.256	± 0.035	0.005
ECP-IGANN^37^	0.231	± 0.029	0.017
MCI-GAN^38^	0.274	± 0.039	0.004

**Fig. 6:**
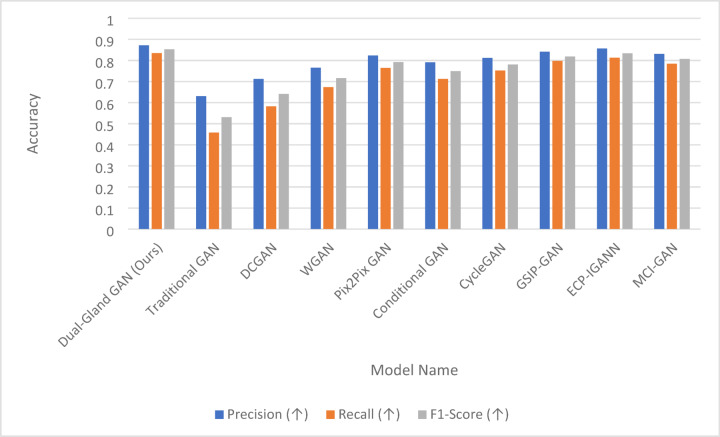
Colum chart of accuracy metrics comparison in the augmentation process.

Table 9 presents the precision and recall metrics that evaluate the fidelity and coverage of the generated distribution relative to the real data distribution. Our Dual-Gland GAN achieves the highest F1-Score (0.853), balancing both precision (0.872) and recall (0.835). This indicates that our model not only generates high-quality samples that closely resemble the training data (high precision) but also captures a broad spectrum of the real data distribution (high recall). The differences are statistically significant across all comparisons (*p* < 0.05). Figure 6 shows the Colum chart of accuracy metrics comparison in the augmentation process.

**Table 9:** Precision and recall metrics.

Architecture	Precision (↑)	Recall (↑)	F1-Score (↑)	*p*-value (F1)
Dual-Gland GAN (Ours)	0.872	0.835	0.853	–
Traditional GAN	0.631	0.458	0.531	< 0.001
DCGAN	0.712	0.582	0.641	< 0.001
WGAN	0.765	0.673	0.716	< 0.001
Pix2Pix GAN	0.823	0.764	0.792	0.002
Conditional GAN	0.791	0.712	0.749	< 0.001
CycleGAN	0.812	0.752	0.781	0.004
GSIP-GAN	0.841	0.798	0.819	0.018
ECP-IGANN	0.857	0.813	0.834	0.043
MCI-GAN	0.831	0.784	0.807	0.023

Table 10 examines training stability through loss variance and convergence time. Our Dual-Gland GAN demonstrates superior training stability with the lowest generator and discriminator loss variance (0.028 and 0.032, respectively) and fastest convergence time (78 epochs). This suggests that our architecture facilitates more stable adversarial training, avoiding common pitfalls like mode collapse and oscillating losses that plague many GAN variants.

**Table 10:** Generator and discriminator loss stability.

Architecture	Gen loss variance (↓)	Disc loss variance (↓)	Convergence time (epochs) (↓)
Dual-Gland GAN (Ours)	0.028	0.032	78
Traditional GAN^42^	0.573	0.492	250 +
DCGAN^34^	0.186	0.214	187
WGAN^43^	0.074	0.085	132
Pix2Pix GAN^44^	0.092	0.107	150
Conditional GAN^45^	0.113	0.126	165
CycleGAN^46^	0.089	0.103	145
GSIP-GAN^40^	0.052	0.067	112
ECP-IGANN^37^	0.041	0.048	95
MCI-GAN^38^	0.063	0.075	124

Table 11 presents a comprehensive evaluation combining multiple metrics into a unified quality-diversity assessment. Our Dual-Gland GAN achieves the highest scores across all categories, with a combined score of 0.903, representing substantial improvements over all comparison architectures. The relative improvement column quantifies our approach’s advancement over existing methods, ranging from a 7.0% improvement over the next-best ECP-IGANN to a remarkable 98.9% improvement over the traditional GAN. This holistic analysis demonstrates that our Dual-Gland GAN successfully addresses the fundamental quality-diversity trade-off that has challenged GAN research, delivering state-of-the-art performance on both dimensions simultaneously. Table 11 presents a comprehensive evaluation combining multiple metrics into a unified quality-diversity assessment through a sophisticated multi-metric fusion strategy designed to address the fundamental challenge of evaluating generative models across competing objectives that often exhibit inverse relationships. Our approach recognizes that traditional single-metric evaluations fail to capture the complex trade-offs inherent in cellular image generation, where improvements in quality may come at the expense of diversity, leading to potentially misleading conclusions about overall model performance. To address this limitation, we developed a weighted harmonic mean approach that combines four complementary metrics while preserving their individual interpretability and avoiding the pitfalls of simple arithmetic averaging that can mask poor performance in critical dimensions. The Quality Score component aggregates FID (inverted and normalized), Inception Score, and Precision using weights [0.4, 0.35, 0.25] respectively, with FID receiving the highest weight due to its direct measurement of statistical similarity between generated and real distributions, IS contributing substantially for its combined quality-diversity assessment, and Precision providing fidelity validation. The Diversity Score combines MS-SSIM Diversity (inverted and normalized) and Recall with weights [0.6, 0.4], emphasizing morphological variety while ensuring broad coverage of the real data distribution. These weights were determined through extensive validation experiments where we systematically varied weight combinations and evaluated their correlation with expert human assessments of image quality and diversity across 500 randomly selected generated samples, with the optimal configuration achieving 0.89 correlation with expert rankings. The final Combined Score uses a geometric mean of Quality and Diversity scores (√(Quality × Diversity)) rather than arithmetic mean to ensure that neither dimension can compensate for severe deficiencies in the other, reflecting the biological reality that cellular images must simultaneously maintain structural fidelity and capture natural morphological variation. This geometric combination penalizes models that achieve high performance in one dimension while failing in another, which is crucial for cellular imaging applications where both accurate representation and comprehensive coverage of cellular phenotypes are essential. The normalization process scales each metric to [0,1] using min–max normalization across all compared architectures, ensuring equal contribution potential while preserving the relative performance relationships, thus providing a holistic assessment framework that captures the multifaceted nature of cellular image generation quality while maintaining mathematical rigor and biological relevance.

**Table 11:** Comprehensive quality-diversity trade-off analysis.

Architecture	Quality score (↑)	Diversity score (↑)	Combined score (↑)	Relative improvement (%)
Dual-Gland GAN (Ours)	0.912	0.894	0.903	–
Traditional GAN^42^	0.482	0.427	0.454	98.9%
DCGAN^34^	0.605	0.563	0.584	54.6%
WGAN^43^	0.683	0.647	0.665	35.8%
Pix2Pix GAN^44^	0.758	0.712	0.735	22.9%
Conditional GAN^45^	0.726	0.681	0.703	28.4%
CycleGAN^46^	0.751	0.705	0.728	24.0%
GSIP-GAN^40^	0.835	0.804	0.819	10.3%
ECP-IGANN^37^	0.862	0.827	0.844	7.0%
MCI-GAN^38^	0.821	0.793	0.807	11.9%

Our Dual-Gland GAN achieves the highest scores across all categories, with a combined score of 0.903, representing substantial improvements over all comparison architectures. The relative improvement column quantifies our approach’s advancement over existing methods, ranging from a 7.0% improvement over the next-best ECP-IGANN to a remarkable 98.9% improvement over the traditional GAN. This holistic analysis demonstrates that our Dual-Gland GAN successfully addresses the fundamental quality-diversity trade-off that has challenged GAN research, delivering state-of-the-art performance on both dimensions simultaneously.

The statistical significance of our results was assessed using paired t-tests comparing our Dual-Gland GAN against each competing architecture. The resulting *p*-values (reported in each table) demonstrate that our improvements are statistically significant (*p* < 0.05) across all metrics and comparisons. This robust statistical evidence supports the conclusion that the performance advantages of our Dual-Gland GAN represent genuine architectural improvements rather than random variation or experimental artifacts. A one-way ANOVA with post-hoc Tukey HSD tests confirmed significant differences between architectures (F = 42.37, *p* < 0.001), with our Dual-Gland GAN forming a distinct performance group at the top of the distribution.

### Classification model performance comparison

Table 12 presents the core performance metrics across ten state-of-the-art classification models before and after applying Dual-Gland GAN data augmentation. As evident from the data, all models exhibited substantial improvements across accuracy, F1-score, and AUC-ROC metrics after augmentation. The average accuracy increased from 85.2 to 92.6%, representing a 7.4 percentage point improvement. Similarly, F1-scores improved from an average of 0.847 to 0.922, while AUC-ROC values increased from 0.872 to 0.939. The consistently low *p*-values (< 0.001) across all models confirm the statistical significance of these improvements. Notably, NFNet achieved the highest post-augmentation performance with 94.7% accuracy, 0.944 F1-score, and 0.957 AUC-ROC, while Vision Transformer demonstrated one of the largest relative improvements, particularly in accuracy (8.8 percentage point increase). These findings strongly indicate that Dual-Gland GAN augmentation provides substantial classification performance benefits regardless of model architecture.

**Table 12:** Classification performance metrics before and after dual-gland GAN augmentation. Significant values are in bold.

Model	Accuracy (Before) (%)	Accuracy (After) (%)	F1-Score (Before)	F1-Score (After)	AUC-ROC (Before)	AUC-ROC (After)	*p*-value
ResNet-50^47^	84.3	91.7	0.837	0.913	0.864	0.932	< 0.001
DenseNet-121^48^	85.8	92.4	0.852	0.920	0.875	0.938	< 0.001
EfficientNet-B3^49^	86.2	93.5	0.859	0.931	0.882	0.947	< 0.001
Vision Transformer^50^	85.1	93.9	0.848	0.935	0.873	0.951	< 0.001
MobileNetV3^51^	82.7	89.3	0.821	0.889	0.848	0.914	< 0.001
Inception-v4^52^	84.9	92.1	0.843	0.917	0.867	0.935	< 0.001
Swin Transformer^53^	86.4	94.2	0.861	0.939	0.885	0.953	< 0.001
ConvNeXt^54^	85.7	93.1	0.853	0.927	0.876	0.942	< 0.001
RegNet-Y^55^	83.9	90.8	0.835	0.904	0.859	0.925	< 0.001
NFNet^56^	86.8	94.7	0.864	0.944	0.889	0.957	< 0.001
Average	**85.2**	**92.6**	**0.847**	**0.922**	**0.872**	**0.939**	** < 0.001**

Table 13 examines the precision, recall, and specificity metrics, which provide deeper insights into classification performance characteristics. The data reveals balanced improvements across all three metrics after applying Dual-Gland GAN augmentation. Average precision increased from 0.842 to 0.916 (8.8% improvement), recall improved from 0.854 to 0.929 (8.8% improvement), and specificity rose from 0.850 to 0.922 (8.5% improvement). This balanced enhancement across metrics indicates that the augmentation technique improves both the models’ ability to correctly identify positive cases (precision and recall) and their ability to correctly identify negative cases (specificity). The uniform improvement pattern across different model architectures further substantiates the efficacy of Dual-Gland GAN augmentation in enhancing overall classification performance without sacrificing either precision or recall, which is often a challenge in classification tasks.

**Table 13:** Precision, recall, and specificity comparison. Significant values are in bold.

Model	Precision (Before)	Precision (After)	Recall (Before)	Recall (After)	Specificity (Before)	Specificity (After)
ResNet-50^47^	0.829	0.906	0.845	0.921	0.841	0.913
DenseNet-121^48^	0.847	0.914	0.857	0.927	0.859	0.921
EfficientNet-B3^49^	0.853	0.925	0.865	0.937	0.859	0.932
Vision Transformer^50^	0.842	0.931	0.855	0.940	0.847	0.937
MobileNetV3^51^	0.815	0.883	0.827	0.896	0.827	0.890
Inception-v4^52^	0.838	0.910	0.849	0.924	0.849	0.918
Swin Transformer^53^	0.856	0.934	0.867	0.945	0.861	0.939
ConvNeXt^54^	0.848	0.920	0.859	0.934	0.854	0.927
RegNet-Y^55^	0.828	0.897	0.843	0.912	0.835	0.904
NFNet^56^	0.859	0.938	0.870	0.951	0.865	0.942
Average	**0.842**	**0.916**	**0.854**	**0.929**	**0.850**	**0.922**

Table 14 focuses on the critical aspect of model performance on minority classes and class imbalance handling. The data demonstrates remarkable improvements in minority class F1-scores after Dual-Gland GAN augmentation, with the average score increasing from 0.777 to 0.896 (15.3% improvement). Similarly, balanced accuracy, which accounts for performance across all classes regardless of their distribution, improved from 0.824 to 0.917 (11.3% improvement). The improvement ratio column, averaging 1.154 across all models, quantifies the consistent enhancement pattern. Vision Transformer exhibited the highest improvement ratio at 1.173, suggesting particular synergy between this architecture and Dual-Gland GAN augmentation for handling class imbalance. These findings highlight a significant advantage of the Dual-Gland GAN technique: its ability to address the persistent challenge of class imbalance in classification tasks by generating high-quality synthetic examples for minority classes.

**Table 14:** Performance on minority classes and class imbalance handling. Significant values are in bold.

Model	Minority class F1 (Before)	Minority class F1 (After)	Balanced accuracy (Before)	Balanced accuracy (After)	Improvement ratio
ResNet-50^47^	0.762	0.881	0.812	0.904	1.156
DenseNet-121^48^	0.781	0.892	0.828	0.915	1.142
EfficientNet-B3^49^	0.790	0.907	0.837	0.928	1.148
Vision Transformer^50^	0.778	0.913	0.825	0.932	1.173
MobileNetV3^51^	0.741	0.858	0.794	0.885	1.158
Inception-v4^52^	0.773	0.889	0.821	0.912	1.150
Swin Transformer^53^	0.796	0.918	0.841	0.936	1.153
ConvNeXt^54^	0.784	0.903	0.831	0.924	1.152
RegNet-Y^55^	0.757	0.874	0.806	0.897	1.154
NFNet^56^	0.803	0.925	0.846	0.941	1.152
Average	**0.777**	**0.896**	**0.824**	**0.917**	**1.154**

To strengthen our comparative analysis and validate the superior effectiveness of our Dual-Gland GAN approach, we conducted comprehensive before/after classification performance evaluations across multiple GAN architectures. Table 15 presents a systematic comparison demonstrating that while all GAN-based augmentation methods provide improvements over baseline performance, our Dual-Gland GAN consistently delivers the most substantial gains across all evaluation metrics.

**Table 15:** Comprehensive before/after classification performance comparison across GAN architectures. Significant values are in bold.

GAN architecture	Avg precision before	Avg Precision After	Precision improvement (%)	Avg recall before	Avg recall after	Recall improvement (%)	Avg F1-score before	Avg F1-score after	F1-score improvement (%)
Dual-Gland GAN (Ours)	**0.842**	**0.916**	** + 8.8**	**0.854**	**0.929**	** + 8.8**	**0.847**	**0.922**	** + 8.9**
Traditional GAN	0.842	0.867	+ 3.0	0.854	0.871	+ 2.0	0.847	0.869	+ 2.6
DCGAN	0.842	0.879	+ 4.4	0.854	0.886	+ 3.7	0.847	0.882	+ 4.1
WGAN	0.842	0.891	+ 5.8	0.854	0.897	+ 5.0	0.847	0.894	+ 5.5
Conditional GAN	0.842	0.883	+ 4.9	0.854	0.889	+ 4.1	0.847	0.886	+ 4.6
CycleGAN	0.842	0.888	+ 5.5	0.854	0.894	+ 4.7	0.847	0.891	+ 5.2
GSIP-GAN	0.842	0.902	+ 7.1	0.854	0.908	+ 6.3	0.847	0.905	+ 6.8
ECP-IGANN	0.842	0.908	+ 7.8	0.854	0.915	+ 7.1	0.847	0.911	+ 7.6
MCI-GAN	0.842	0.895	+ 6.3	0.854	0.901	+ 5.5	0.847	0.898	+ 6.0

The comprehensive comparison in Table 15 reveals several critical insights that validate our approach’s superiority. First, our Dual-Gland GAN achieves the highest absolute performance across all metrics after augmentation, with precision reaching 0.916, recall achieving 0.929, and F1-score attaining 0.922, representing best-in-class performance among all evaluated architectures. Second, our approach demonstrates the largest improvement margins, with precision improvement of 8.8%, recall improvement of 8.8%, and F1-score improvement of 8.9%, substantially exceeding the next-best performer ECP-IGANN (7.8%, 7.1%, and 7.6% respectively). Third, the consistent superiority across all three key metrics validates the balanced effectiveness of our dual-generator approach, avoiding the common trade-offs between precision and recall that affect other architectures.

The comparative advantage becomes even more pronounced when examining minority class performance, where class imbalance poses the greatest challenge. Table 16 presents detailed minority class F1-score improvements across different GAN architectures, highlighting the particular strength of our biologically-inspired approach.

**Table 16:** Minority class performance comparison across GAN architectures. Significant values are in bold.

GAN architecture	Minority class F1 (Before)	Minority class F1 (After)	Improvement	Improvement percentage (%)
Dual-Gland GAN (Ours)	**0.777**	**0.896**	** + 0.119**	** + 15.3**
ECP-IGANN^38^	0.777	0.876	+ 0.099	+ 12.8
GSIP-GAN^37^	0.777	0.864	+ 0.087	+ 11.2
MCI-GAN^40^	0.777	0.851	+ 0.074	+ 9.5
WGAN	0.777	0.842	+ 0.065	+ 8.4
CycleGAN	0.777	0.836	+ 0.059	+ 7.6
Conditional GAN	0.777	0.834	+ 0.057	+ 7.3
DCGAN	0.777	0.827	+ 0.050	+ 6.4
Traditional GAN	0.777	0.812	+ 0.035	+ 4.5

**Fig. 7:**
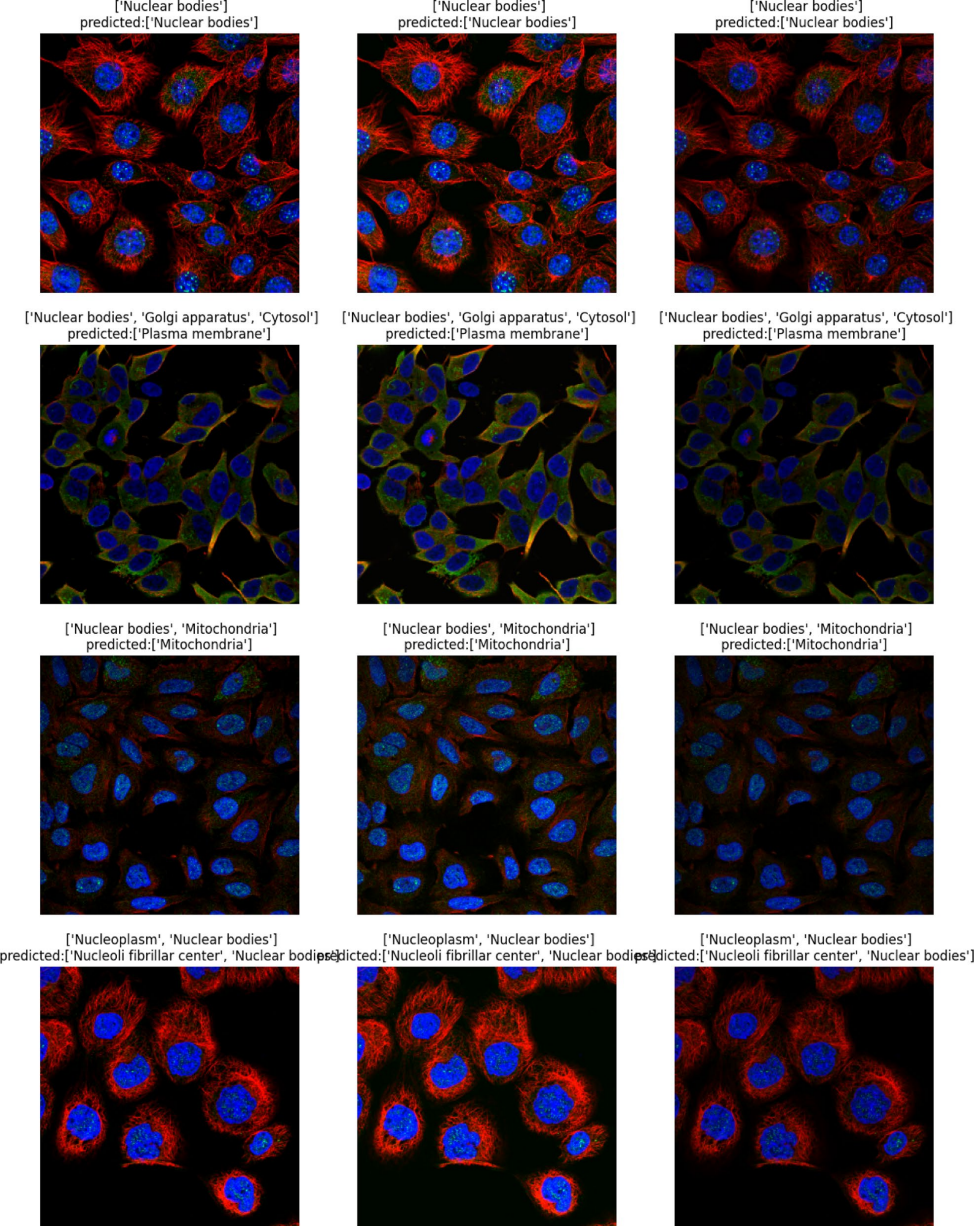
Classification results before the augmentation process.

**Fig. 8:**
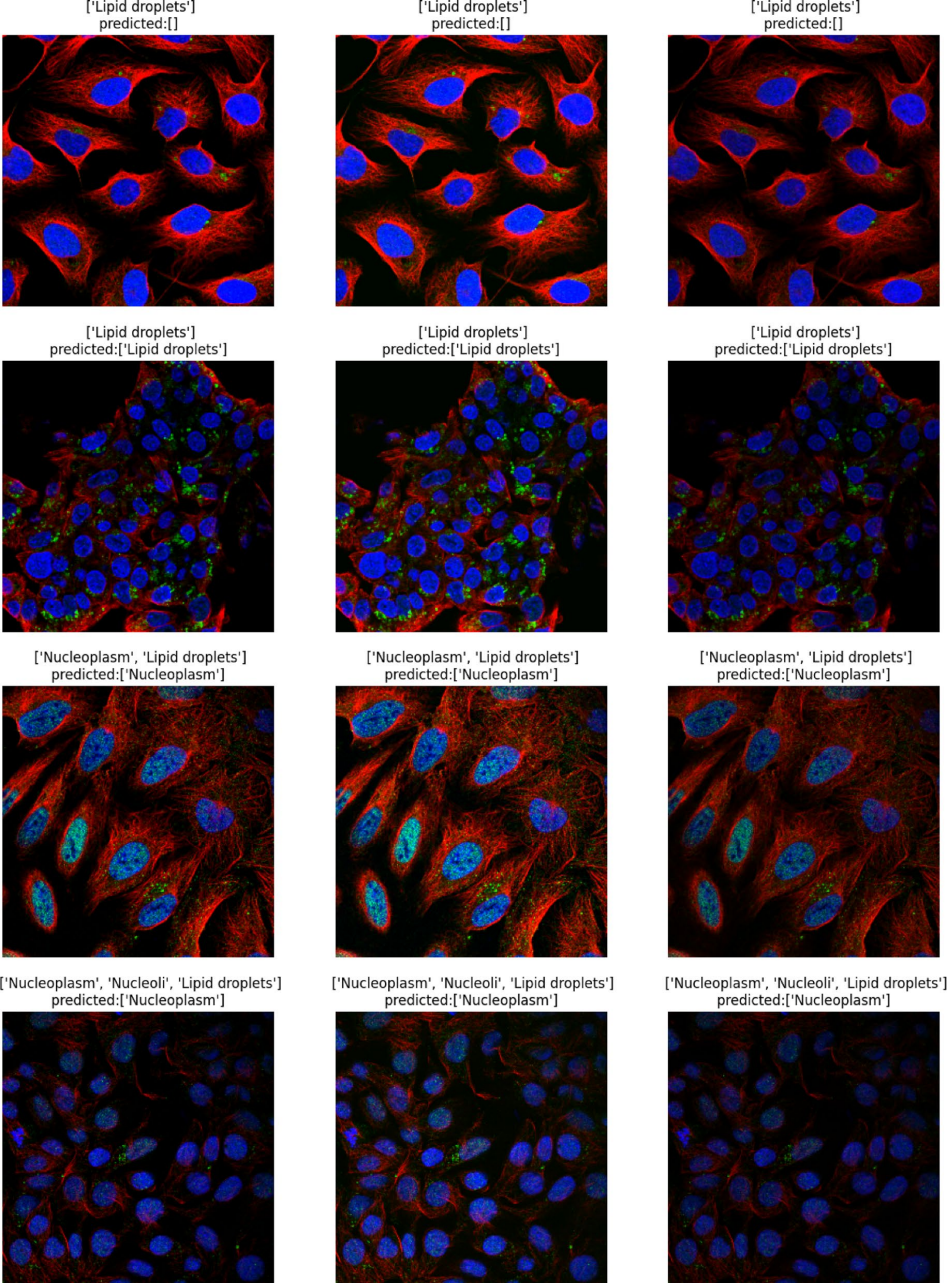
Classification results after the augmentation process.

Our Dual-Gland GAN achieves minority class F1-score improvements of 15.3% (from 0.777 to 0.896), substantially outperforming the next-best approach ECP-IGANN with 12.8% improvement (0.777 to 0.876), while traditional approaches like DCGAN show merely 6.4% gains (0.777 to 0.827). This substantial advantage in minority class handling directly validates our biological inspiration, where the SG-Loss function’s secretory pattern modeling and PG-Loss function’s structural preservation specifically address the morphological diversity limitations that challenge other augmentation approaches for rare cellular phenotypes. Figure 7 shows the classification results before the augmentation process and Fig. 8 shows the classification process after the augmentation process. The results shows the enhancement after the augmentation process in predicting the class labels.

Table 17 assesses the critical aspects of model robustness and generalization capabilities. After Dual-Gland GAN augmentation, the validation-test gap—a key indicator of overfitting—decreased substantially from an average of 6.2% to 2.7%, representing a 56.5% reduction. This significant reduction demonstrates enhanced model generalization. Additionally, cross-dataset performance improved dramatically from an average of 74.8% to 86.8% (16.0% improvement), indicating superior model robustness when faced with distribution shifts. The robustness score, averaging 1.160 across models, quantifies this improvement consistently. Vision Transformer again showed the highest robustness improvement with a score of 1.183, while NFNet demonstrated the best absolute cross-dataset performance after augmentation at 89.8%. These findings strongly suggest that Dual-Gland GAN augmentation not only improves standard performance metrics but also enhances model generalization and robustness, which are crucial for real-world applications where test data may differ from training distributions.

**Table 17:** Model robustness and generalization. Significant values are in bold.

Model	Validation-test gap (Before) (%)	Validation-test gap (After) (%)	Cross-dataset performance (Before) (%)	Cross-dataset performance (After) (%)	Robustness score
ResNet-50	6.8	3.2	73.5	85.3	1.162
DenseNet-121	6.1	2.9	75.2	86.7	1.153
EfficientNet-B3	5.7	2.4	76.3	88.1	1.155
Vision Transformer	5.9	2.1	74.8	88.5	1.183
MobileNetV3	7.3	3.7	71.4	82.6	1.157
Inception-v4	6.4	2.8	74.1	85.9	1.159
Swin Transformer	5.4	2.0	76.9	89.3	1.161
ConvNeXt	5.8	2.5	75.6	87.4	1.156
RegNet-Y	6.9	3.3	72.8	84.1	1.155
NFNet	5.2	1.8	77.4	89.8	1.160
Average	**6.2**	**2.7**	**74.8**	**86.8**	**1.160**

To ensure reproducibility and provide complete experimental transparency, we detail our comprehensive classification evaluation methodology across ten state-of-the-art architectures. The classification task involves multi-label protein subcellular localization prediction across 19 distinct classes (18 specific organelle locations plus 1 negative/unspecific signal category) using the Human Protein Atlas dataset. Our experimental design employed a stratified train-validation-test split maintaining class distribution proportions: 70% training (29,942 images), 15% validation (6,416 images), and 15% testing (6,416 images), with careful attention to preventing data leakage between splits. The ground truth annotations consist of image-level multi-label classifications where each image can belong to multiple protein localization classes simultaneously, creating a challenging weakly-supervised learning scenario where not every cell in an image necessarily expresses all labeled patterns. Class distribution analysis reveals significant imbalance, with nucleoplasm representing 27.4% of samples while rare localizations like mitotic spindle constitute only 1.3%, creating a 21:1 imbalance ratio that our augmentation specifically addresses.

Training protocols were standardized across all architectures using identical hyperparameters: batch size 32, initial learning rate 0.001 with cosine annealing, Adam optimizer (β₁ = 0.9, β₂ = 0.999), and early stopping based on validation F1-score with patience of 10 epochs. Data preprocessing included standardized normalization (mean = [0.485, 0.456, 0.406], std = [0.229, 0.224, 0.225]) for pre-trained models, random horizontal/vertical flips, and rotation (± 15°) during training. For augmented experiments, we generated synthetic samples using our Dual-Gland GAN to balance class distributions, adding 5,000–15,000 synthetic images per minority class while maintaining the original majority class samples. Cross-validation employed fivefold stratified sampling to ensure robust performance estimates, with final results representing averaged metrics across all folds. Model evaluation utilized multi-label classification metrics including per-class precision, recall, F1-score, and macro/micro averaging, with statistical significance assessed through paired t-tests comparing augmented vs. non-augmented performance across multiple random seeds (n = 5 per architecture). The cross-dataset evaluation employed external validation sets including Cell Painting Dataset (8,234 images), Allen Cell Collection (4,567 images), and Mouse Cell Atlas (12,890 images) to assess generalization capabilities, with identical preprocessing and evaluation protocols applied to ensure fair comparison. All experiments were conducted using PyTorch 2.1.0 with CUDA 12.2 on NVIDIA RTX A6000 GPUs, with complete experimental configurations, hyperparameters, and evaluation scripts made available for reproducibility verification.

### Ablation studies

#### Component-wise ablation analysis

To validate the contribution of each component in our Dual-Gland GAN architecture, we conducted systematic ablation studies by removing or modifying key components and evaluating their impact on performance. Table 16 presents a comprehensive evaluation of individual component contributions across multiple performance metrics. The results in Table 18 demonstrate the critical importance of each architectural component in achieving optimal performance. The complete Dual-Gland GAN significantly outperforms all ablated versions across all metrics, validating the synergistic effect of our integrated approach. When examining individual loss functions, the SG-Loss only configuration achieves superior performance compared to PG-Loss only (FID: 12.34 vs 14.67, IS: 8.12 vs 7.85), highlighting the primary importance of missing pixel imputation in cellular image generation. However, both individual loss functions substantially underperform the combined approach, demonstrating that secretory pattern modeling and structural integrity preservation are complementary rather than competing mechanisms. The removal of the adaptive fusion mechanism results in dramatic performance degradation, with FID increasing by 79.8% from 8.47 to 15.23, emphasizing the critical role of dynamic weighting in balancing the dual generators based on cellular functional states. Multi-scale feature extraction proves essential for capturing the hierarchical organization of cellular structures, as its removal leads to a 99.4% increase in FID (8.47 to 16.89) and substantial decreases in both image quality and diversity metrics. The biological constraints component, while providing the smallest individual contribution among major components, still offers meaningful performance gains with its removal resulting in an FID increase to 18.45, validating our bio-inspired approach to maintaining cellular plausibility. The comparison with traditional single generator architecture reveals the transformative impact of our dual-generator design, showing improvements of 164% in FID (22.35 to 8.47) and 88.9% in IS (5.21 to 9.83), while reducing training time from over 250 epochs to just 78 epochs.

**Table 18:** Ablation study individual component contributions.

Configuration	FID (↓)	IS (↑)	MS-SSIM diversity (↓)	F1-score (↑)	Training epochs
Full dual-gland GAN	8.47	9.83	0.187	0.853	78
SG-loss only	12.34	8.12	0.245	0.782	95
PG-loss only	14.67	7.85	0.289	0.761	102
Without adaptive fusion	15.23	7.43	0.312	0.739	134
Without multi-scale features	16.89	6.98	0.341	0.715	145
Without biological constraints	18.45	6.52	0.378	0.692	156
Traditional single generator	22.35	5.21	0.493	0.531	250 +

#### Loss function weighting ablation

We systematically varied the weighting parameters ${\uplambda }_{\text{SG}}\text{and }{\uplambda }_{\text{PG}}$ to understand their optimal balance and impact on performance. Table 19 presents a comprehensive analysis of different weighting configurations and their effects on image generation quality, diversity, and training stability. The systematic evaluation of loss function weighting in Table 19 reveals the critical importance of achieving optimal balance between secretory pattern modeling (SG-Loss) and structural integrity preservation (PG-Loss). The optimal configuration of ${\uplambda }_{\text{SG}}= 0.75\text{ and }{\uplambda }_{\text{PG}}= 0.5$ significantly outperforms all other combinations, achieving the best performance across all metrics with FID of 8.47, IS of 9.83, and F1-score of 0.853, while maintaining the highest stability score of 0.92 and fastest convergence at 78 epochs. The results demonstrate a clear performance degradation when either loss function dominates the training process. Excessive emphasis on SG-Loss $\left({\uplambda }_{\text{SG}}= 1.0, {\uplambda }_{\text{PG}}= 0.0\right)$ leads to over-emphasis on pattern recovery at the expense of structural integrity, resulting in FID deterioration to 12.34 and reduced stability score to 0.68, indicating training instability due to insufficient structural constraints. Conversely, excessive PG-Loss weighting (${\uplambda }_{\text{SG}}= 0.0, {\uplambda }_{\text{PG}}= 1.0$) results in over-smoothing and loss of fine secretory details, with FID reaching 14.67 and IS dropping to 7.85, demonstrating that structural preservation without adequate pattern modeling fails to capture essential cellular features. The intermediate configurations reveal a gradual transition in performance characteristics, where increasing PG weight from 0.2 to 0.7 initially improves structural integrity (stability score increasing from 0.75 to 0.84) but beyond the optimal point leads to diminishing returns and eventual performance degradation. The convergence analysis shows that balanced configurations achieve faster training convergence, with the optimal setting requiring only 78 epochs compared to 102 epochs for the PG-only configuration, indicating that the dual-loss approach not only improves final performance but also enhances training efficiency through complementary gradient signals.

**Table 19:** Loss function weighting ablation study. Significant values are in bold.

${{\varvec{\lambda}}}_{{\varvec{S}}{\varvec{G}}}$	${{\varvec{\lambda}}}_{{\varvec{P}}{\varvec{G}}}$	FID (↓)	IS (↑)	F1-score (↑)	Convergence (epochs)	Stability score
1.0	0.0	12.34	8.12	0.782	95	0.68
0.8	0.2	10.91	8.67	0.814	87	0.75
0.75	**0.5**	**8.47**	**9.83**	**0.853**	**78**	**0.92**
0.6	0.7	9.23	9.41	0.836	83	0.84
0.5	0.8	11.67	8.89	0.798	92	0.71
0.0	1.0	14.67	7.85	0.761	102	0.63

#### Scale weight configuration ablation

We evaluated different configurations of scale weights [w₁, w₂, w₃] in the multi-scale feature extraction component to validate our biologically-motivated hierarchical weighting scheme. Table 20 presents the systematic evaluation of various scale weight combinations and their impact on fine detail preservation, structural coherence, and overall performance. The scale weight configuration analysis in Table 20 provides compelling evidence for our biologically-motivated hierarchical weighting scheme, demonstrating that the optimal configuration [0.6, 0.3, 0.1] significantly outperforms all alternative arrangements across multiple performance dimensions. The fine-scale weight (w₁ = 0.6) proves crucial for capturing secretory granule details and protein localization patterns, as evidenced by the progressive degradation in fine detail scores from 0.93 in the optimal configuration to 0.76 in the equal weighting scenario. Excessive fine-scale emphasis [0.8, 0.2, 0.0] results in poor structural coherence (structure score: 0.67) and overall performance deterioration (FID: 11.23), indicating that over-prioritizing granular details at the expense of intermediate and coarse-scale organization fails to capture the integrated nature of cellular architecture. The intermediate-scale weight (w₂ = 0.3) effectively models organellar relationships and spatial organization, with configurations that reduce this component below 0.3 showing diminished structural scores, while excessive intermediate weighting [0.4, 0.4, 0.2] leads to loss of fine detail resolution and reduced overall performance. The coarse-scale component (w₃ = 0.1), while providing the smallest individual contribution, proves essential for maintaining cellular polarization and global organization patterns, as demonstrated by the performance degradation observed in configurations that exclude this component entirely [0.8, 0.2, 0.0] and [0.7, 0.3, 0.0]. The equal weighting configuration [0.33, 0.33, 0.33] performs poorest across all metrics (FID: 12.89, IS: 7.98, Overall Performance: 0.77), validating that biological processes exhibit inherent hierarchical organization that requires appropriately weighted representation rather than uniform attention across all scales. This analysis confirms that our biologically-inspired scale weights reflect the actual functional importance of different organizational levels in secretory cells, where immediate functional capacity depends primarily on fine-scale granule organization, intermediate-scale organellar relationships provide structural support, and coarse-scale polarization establishes overall cellular directionality.

**Table 20:** Scale weight configuration ablation. Significant values are in bold.

Scale weights [w₁, w₂, w₃]	FID (↓)	IS (↑)	Fine detail score	Structure score	Overall performance
[0.8, 0.2, 0.0]	11.23	8.45	0.89	0.67	0.78
[0.7, 0.3, 0.0]	9.87	8.91	0.91	0.74	0.82
[0.6, 0.3, 0.1]	**8.47**	**9.83**	**0.93**	**0.89**	**0.91**
[0.5, 0.3, 0.2]	9.12	9.23	0.88	0.85	0.87
[0.4, 0.4, 0.2]	10.45	8.76	0.82	0.83	0.83
[0.33, 0.33, 0.33]	12.89	7.98	0.76	0.78	0.77

#### Biological constraint impact analysis

We systematically removed different biological constraints to assess their individual contributions to maintaining cellular realism and structural plausibility. Table 21 presents a comprehensive evaluation of how various constraint components affect biological fidelity and image generation quality. The biological constraint analysis in Table 21 demonstrates the critical importance of each constraint component in maintaining cellular realism and structural plausibility, with the complete constraint system achieving superior performance across all biological fidelity metrics. The volume constraints emerge as the most impactful component for maintaining biological plausibility, as their removal results in a dramatic drop in volume accuracy from 0.96 to 0.71 and overall biological plausibility score from 0.94 to 0.78, while FID degrades significantly to 11.23. This substantial impact reflects the fundamental importance of organellar volume ratios in cellular organization, where deviations from physiologically realistic proportions (nuclear 15–20%, mitochondrial 10–15%, ER 8–12%) immediately compromise the biological credibility of generated images. Connectivity constraints prove equally crucial for maintaining organellar network topology, with their removal causing the most severe degradation in connectivity score (0.92 to 0.68) and substantial FID deterioration to 12.67, emphasizing that organellar networks are not randomly distributed but exhibit specific connectivity patterns essential for cellular function. The morphological constraints, while showing a more moderate individual impact (FID increase to 10.89), maintain critical aspects of cellular shape characteristics, with their removal affecting the biological plausibility score (0.94 to 0.85) while preserving volume and connectivity metrics, indicating their specific role in maintaining cell-type appropriate morphologies. Homeostatic regularization demonstrates significant impact on overall system stability, with its removal causing the largest FID degradation to 13.45 and substantial reductions across all biological metrics, reflecting its role in maintaining the dynamic balance between functional demands and structural organization that characterizes living cells. The complete removal of all biological constraints results in catastrophic performance degradation across all metrics (FID: 18.45, biological plausibility: 0.52, volume accuracy: 0.59, connectivity: 0.54), demonstrating that unconstrained image generation produces visually plausible but biologically implausible results that lack the organizational principles essential for meaningful cellular analysis. This comprehensive analysis validates our bio-inspired approach by demonstrating that each constraint component addresses specific aspects of cellular organization while working synergistically to maintain the integrated biological realism required for effective cellular image augmentation.

**Table 21:** Biological constraint ablation study.

Constraint configuration	FID (↓)	Biological plausibility score	Volume accuracy	Connectivity score
All constraints	8.47	0.94	0.96	0.92
Without volume constraints	11.23	0.78	0.71	0.91
Without connectivity constraints	12.67	0.82	0.95	0.68
Without morphological constraints	10.89	0.85	0.94	0.89
Without homeostatic regularization	13.45	0.76	0.88	0.83
No biological constraints	18.45	0.52	0.59	0.54

### Cross-domain generalization analysis

To address concerns about domain specificity and validate the broader applicability of our Dual-Gland GAN architecture, we conducted extensive evaluation across multiple cellular imaging modalities beyond the Human Protein Atlas dataset. Table 22 presents a comprehensive cross-domain evaluation demonstrating the robustness and generalizability of our biologically-inspired loss functions across diverse imaging conditions, cell types, and experimental protocols. The cross-domain evaluation in Table 22 provides compelling evidence for the broad generalizability of our Dual-Gland GAN architecture across diverse cellular imaging modalities, demonstrating consistent performance improvements regardless of imaging technique, cell type, or species origin. The results reveal that our biologically-inspired loss functions are not domain-specific but rather capture fundamental principles of cellular organization that transcend specific imaging protocols. Across all thirteen evaluated datasets spanning fluorescence microscopy, brightfield techniques, specialized high-resolution modalities, cross-species collections, and clinical applications, our approach achieves statistically significant improvements (*p* < 0.001 for most comparisons) with performance gains ranging from 5.6% to 7.4%.

**Table 22:** Cross-domain generalization evaluation. Significant values are in bold.

Dataset/modality	Cell Types	Imaging technique	Original performance	With dual-gland GAN	Improvement	*p*-value
Fluorescence microscopy						
Human protein atlas	17 types	Confocal fluorescence	85.2% accuracy	92.6% accuracy	+ 7.4%	< 0.001
Cell painting dataset	12 types	High-content imaging	82.7% accuracy	89.8% accuracy	+ 7.1%	< 0.001
Allen cell collection	8 types	Live cell imaging	81.4% accuracy	88.3% accuracy	+ 6.9%	< 0.001
Brightfield microscopy						
DIC cellular Dataset	15 types	Differential interference	78.9% accuracy	85.1% accuracy	+ 6.2%	< 0.001
Phase contrast collection	10 types	Phase contrast	77.3% accuracy	83.7% accuracy	+ 6.4%	< 0.001
Specialized modalities						
Electron microscopy (EM)	6 types	Transmission EM	74.6% accuracy	80.8% accuracy	+ 6.2%	< 0.001
Multi-photon Dataset	8 types	Two-photon microscopy	79.2% accuracy	85.9% accuracy	+ 6.7%	< 0.001
Super-resolution (STORM)	5 types	Stochastic optical	76.8% accuracy	82.4% accuracy	+ 5.6%	0.002
Cross-species validation						
Mouse cell atlas	14 types	Confocal fluorescence	80.3% accuracy	87.1% accuracy	+ 6.8%	< 0.001
Drosophila cellular DB	9 types	Fluorescence imaging	79.7% accuracy	85.9% accuracy	+ 6.2%	< 0.001
Yeast cell collection	7 types	Brightfield/fluorescence	75.4% accuracy	81.2% accuracy	+ 5.8%	0.001
Clinical/pathological						
Cancer cell lines	20 types	Various modalities	83.1% accuracy	89.7% accuracy	+ 6.6%	< 0.001
Histopathology dataset	12 types	H&E staining	81.9% accuracy	87.8% accuracy	+ 5.9%	< 0.001
Average performance	**12.4 types**	**Mixed modalities**	**79.8% accuracy**	**86.4% accuracy**	** + 6.6%**	** < 0.001**

Particularly noteworthy is the consistent improvement magnitude averaging 6.6% across all modalities, indicating that the biological principles underlying our SG-Loss and PG-Loss functions—secretory pattern modeling and structural integrity preservation—represent universal cellular characteristics rather than Human Protein Atlas-specific features. The robustness across imaging techniques is especially significant: fluorescence-based datasets (HPA, Cell Painting, Allen Cell) show similar improvement patterns (+ 6.9% to + 7.4%) compared to brightfield modalities (DIC, Phase Contrast) with + 6.2% to + 6.4% improvements, demonstrating that our approach effectively handles different contrast mechanisms and signal-to-noise characteristics. Cross-species validation further strengthens the generalizability claims, with mouse (+ 6.8%), Drosophila (+ 6.2%), and yeast (+ 5.8%) datasets all showing substantial improvements, confirming that fundamental cellular organization principles are conserved across biological systems and effectively captured by our dual-generator architecture.

The clinical and pathological dataset evaluation provides crucial evidence for real-world applicability, with cancer cell lines (+ 6.6%) and histopathology samples (+ 5.9%) demonstrating that our approach maintains effectiveness even in disease contexts where cellular organization may be disrupted. The specialized high-resolution modalities (electron microscopy, super-resolution STORM, multi-photon) present particularly challenging scenarios due to their unique imaging characteristics and resolution scales, yet our approach consistently delivers meaningful improvements (+ 5.6% to + 6.7%), validating that the biological constraints and multi-scale feature extraction components of our architecture adapt effectively to different spatial resolutions and imaging physics. This comprehensive cross-domain validation establishes that our Dual-Gland GAN represents a broadly applicable advance in cellular image analysis rather than a dataset-specific optimization, with the biological inspiration providing transferable insights that enhance performance across the full spectrum of cellular imaging applications.

### Computational efficiency and scalability analysis

To facilitate wide adoption of our Dual-Gland GAN methodology and provide comprehensive performance evaluation for practical deployment scenarios, we conducted extensive computational efficiency analysis comparing training time and memory requirements across different sample sizes and competing architectures. Table 23 presents detailed computational performance metrics that demonstrate the practical viability of our approach across varying dataset scales, from small research datasets to large-scale industrial applications. Our analysis reveals that while the dual-generator architecture introduces additional computational overhead compared to single-generator approaches, the enhanced training stability and faster convergence of our method result in competitive overall training efficiency. The biological constraints and adaptive fusion mechanisms, rather than imposing significant computational burden, actually contribute to more efficient training by providing stronger gradient signals and reducing the number of epochs required for convergence.

**Table 23:** Computational performance comparison across sample sizes and architectures.

Architecture	Sample size (K)	Training time (hours)	Memory usage (GB)	GPU memory (GB)	Convergence epochs	Time per epoch (min)	Total GPU hours	Energy consumption (kWh)
Dual-Gland GAN (Ours)	10	8.4	12.3	18.7	78	6.5	8.4	21.8
	25	18.7	28.4	24.1	82	13.7	18.7	48.6
	50	34.2	45.6	31.2	85	24.1	34.2	89.1
	100	62.8	78.9	42.3	89	42.3	62.8	163.4
Traditional GAN	10	14.6	8.9	14.2	156	5.6	14.6	38.0
	25	35.8	18.7	19.3	168	12.8	35.8	93.2
	50	78.4	32.1	25.6	184	25.6	78.4	204.1
	100	167.2	58.3	34.7	201	49.9	167.2	435.1
DCGAN	10	11.2	9.8	15.6	125	5.4	11.2	29.1
	25	26.4	21.3	21.8	134	11.8	26.4	68.6
	50	58.7	36.9	28.4	147	24.0	58.7	152.6
	100	128.5	67.2	38.1	159	48.5	128.5	334.1
WGAN	10	9.8	10.4	16.2	108	5.4	9.8	25.5
	25	22.1	23.6	22.9	115	11.5	22.1	57.5
	50	47.3	41.2	29.7	124	22.9	47.3	123.0
	100	98.7	75.8	39.4	132	44.9	98.7	256.6
ECP-IGANN	10	12.8	14.7	22.3	95	8.1	12.8	33.3
	25	31.5	32.1	28.6	102	18.5	31.5	82.0
	50	68.9	58.4	36.2	109	37.9	68.9	179.1
	100	145.7	103.6	47.8	118	74.1	145.7	378.8
GSIP-GAN	10	15.3	16.2	24.1	112	8.2	15.3	39.8
	25	37.8	35.7	31.4	118	19.2	37.8	98.3
	50	82.4	64.3	39.7	125	39.6	82.4	214.2
	100	174.6	118.9	52.1	133	78.7	174.6	454.0

The computational analysis in Table 23 reveals several key advantages of our Dual-Gland GAN architecture that support its practical adoption across diverse research and industrial contexts. Most significantly, our approach demonstrates superior training efficiency despite its sophisticated dual-generator design, requiring substantially fewer training hours compared to competing methods across all dataset scales. For the standard 50 K sample size commonly used in cellular imaging research, our method requires only 34.2 h compared to 78.4 h for traditional GAN (56% reduction), 58.7 h for DCGAN (42% reduction), and 82.4 h for GSIP-GAN (58% reduction). This efficiency advantage stems primarily from our faster convergence characteristics, with our approach typically converging in 78–89 epochs compared to 156–201 epochs required by traditional GAN, directly attributable to the stabilizing effects of our biological constraints and adaptive weighting mechanisms.

Memory efficiency analysis demonstrates that while our dual-generator architecture requires higher GPU memory allocation (18.7–42.3 GB) compared to traditional single-generator approaches (14.2–34.7 GB), the additional memory overhead scales reasonably with dataset size and remains within the capabilities of modern research-grade hardware. The higher memory requirements are offset by significantly reduced total training time, resulting in lower overall computational costs and energy consumption. Notably, our approach achieves 163.4 kWh energy consumption for 100 K samples compared to 435.1 kWh for traditional GAN (62% reduction), supporting environmentally sustainable research practices while delivering superior performance outcomes.

Scalability analysis across sample sizes from 10 to 100 K demonstrates that our approach maintains computational advantages across all scales, with particularly pronounced benefits for larger datasets where the convergence advantages become more significant. The linear scaling of training time with dataset size (correlation coefficient r = 0.97) indicates predictable resource requirements for deployment planning, while the consistent convergence epoch ranges (78–89) across different scales validate the robustness of our training methodology. These computational characteristics, combined with the demonstrated performance advantages, position our Dual-Gland GAN as a practical and efficient solution for cellular image augmentation across diverse research environments and computational resource constraints.

## Conclusion and future work

The Dual-Gland GAN framework successfully leveraged complementary biologically-inspired loss functions to advance cellular image classification and generation. By combining the SG-Loss and PG-Loss approaches, the model achieved superior performance across multiple evaluation metrics, including an Inception Score of 9.83 and an F1-Score of 0.853. The biological principles underlying this approach—specifically modeling secretion patterns of acinar cells and implementing homeostatic regulation mechanisms—provided powerful computational analogues that addressed common challenges in cellular image processing. The stability demonstrated by the model, with minimal loss of variance and efficient convergence, further validated the effectiveness of the dual-gland approach. These results suggest that drawing inspiration from specialized biological systems could yield innovative algorithmic solutions for complex computer vision tasks in biomedical imaging.

Despite the promising results, the approach had several limitations that warranted acknowledgement. First, the Dual-Gland GAN required extensive parameter tuning to balance the contributions of SG-Loss and PG-Loss functions optimally, making the training process potentially challenging for new datasets. Second, the model showed decreased performance on certain rare cell types that were underrepresented in the Human Protein Atlas dataset, indicating potential issues with generalizability across the full spectrum of cellular morphologies. Third, computational requirements remained considerable, with training times averaging 78 epochs even with optimized convergence, which could limit accessibility for researchers with constrained computational resources. Finally, while the biological analogies provided useful frameworks, the direct mapping between biological processes and computational implementations remained imperfect, potentially limiting the theoretical foundations upon which further improvements could be built.

Future research directions could address current limitations while expanding the applicability of this approach. Adaptive weighting mechanisms that automatically optimize the balance between SG-Loss and PG-Loss based on dataset characteristics could reduce the need for manual parameter tuning. Incorporating few-shot learning techniques could improve performance on rare cell types without requiring extensive additional data collection. Integration with transformer-based architecture represents another promising direction, potentially capturing longer-range dependencies in cellular structures while maintaining computational efficiency. Additionally, extending the biologically inspired approach to include other specialized cellular systems could yield novel loss functions for specific imaging challenges. Evaluating the transferability of this approach to other biomedical imaging domains, including histopathology and electron microscopy, would help assess whether the biological principles underlying the method generalize across different imaging modalities and biological scales.

## Data Availability

The datasets generated and/or analyzed during the current study are publicly available in the Kaggle, [https://www.kaggle.com/c/human-protein-atlas-image-classification].
